# Biallelic interferon regulatory factor 8 mutation: A complex immunodeficiency syndrome with dendritic cell deficiency, monocytopenia, and immune dysregulation

**DOI:** 10.1016/j.jaci.2017.08.044

**Published:** 2018-06

**Authors:** Venetia Bigley, Sheetal Maisuria, Urszula Cytlak, Laura Jardine, Matthew A. Care, Kile Green, Merry Gunawan, Paul Milne, Rachel Dickinson, Sarah Wiscombe, David Parry, Rainer Doffinger, Arian Laurence, Claudia Fonseca, Oda Stoevesandt, Andrew Gennery, Andrew Cant, Reuben Tooze, A. John Simpson, Sophie Hambleton, Sinisa Savic, Gina Doody, Matthew Collin

**Affiliations:** a Institute of Cellular Medicine, Newcastle University, Newcastle upon Tyne, United Kingdom; b Newcastle upon Tyne Hospitals NHS Foundation Trust, Newcastle upon Tyne, United Kingdom; c Leeds Institute of Rheumatic and Musculoskeletal Medicine, University of Leeds, Leeds, United Kingdom; d Leeds Institute of Cancer and Pathology, University of Leeds, Leeds, United Kingdom; e Leeds Institute of Biomedical and Clinical Sciences, University of Leeds, Leeds, United Kingdom; h National Institute for Health Research–Leeds Musculoskeletal Biomedical Research Unit and Institute of Rheumatic and Musculoskeletal Medicine, University of Leeds, Leeds, United Kingdom; f Department of Clinical Biochemistry and Immunology, Addenbrookes Hospital, Cambridge, United Kingdom; g Cambridge Protein Arrays, Babraham Research Campus, Cambridge, United Kingdom

**Keywords:** Interferon regulatory factor 8, immunodeficiency, dendritic cell, monocyte, myeloproliferation, interferon, AICE, Activator protein 1–interferon regulatory factor composite element, BAL, Bronchoalveolar lavage, cDC, Conventional dendritic cell, CDR3, Complementarity-determining region 3, DC, Dendritic cell, EICE, Ets–interferon regulatory factor composite element, EMSA, Electrophoretic mobility shift assay, HA, Hemagglutinin, IAD, Interferon regulatory factor–associated domain, IRF, Interferon regulatory factor, ISRE, Interferon-stimulated response element, NK, Natural killer, pDC, Plasmacytoid dendritic cell, SSC, Side scatter, STAT, Signal transducer and activator of transcription, TF, Transcription factor, TLR, Toll-like receptor, Treg, Regulatory T

## Abstract

**Background:**

The homozygous K108E mutation of interferon regulatory factor 8 *(IRF8)* is reported to cause dendritic cell (DC) and monocyte deficiency. However, more widespread immune dysfunction is predicted from the multiple roles ascribed to IRF8 in immune cell development and function.

**Objective:**

We sought to describe the effect on hematopoiesis and immunity of the compound heterozygous R83C/R291Q mutation of *IRF8*, which is present in a patient with recurrent viral infection, granuloproliferation, and intracerebral calcification.

**Methods:**

Variant *IRF8* alleles were identified by means of exome sequencing, and their function was tested by using reporter assays. The cellular phenotype was studied in detail by using flow cytometry, functional immunologic assay transcriptional profiling, and antigen receptor profiling.

**Results:**

Both mutations affected conserved residues, and R291Q is orthologous to R294, which is mutated in the BXH2 IRF8-deficient mouse. R83C showed reduced nuclear translocation, and neither mutant was able to regulate the Ets/IRF composite element or interferon-stimulated response element, whereas R291Q retained BATF/JUN interactions. DC deficiency and monocytopenia were observed in blood, dermis, and lung lavage fluid. Granulocytes were consistently increased, dysplastic, and hypofunctional. Natural killer cell development and maturation were arrested. T_H_1, T_H_17, and CD8^+^ memory T-cell differentiation was significantly reduced, and T cells did not express CXCR3. B-cell development was impaired, with fewer memory cells, reduced class-switching, and lower frequency and complexity of somatic hypermutation. Cell-specific gene expression was widely disturbed in interferon- and IRF8-regulated transcripts.

**Conclusions:**

This analysis defines the clinical features of human biallelic IRF8 deficiency, revealing a complex immunodeficiency syndrome caused by DC and monocyte deficiency combined with widespread immune dysregulation.

## Graphical abstract

**Figure:**
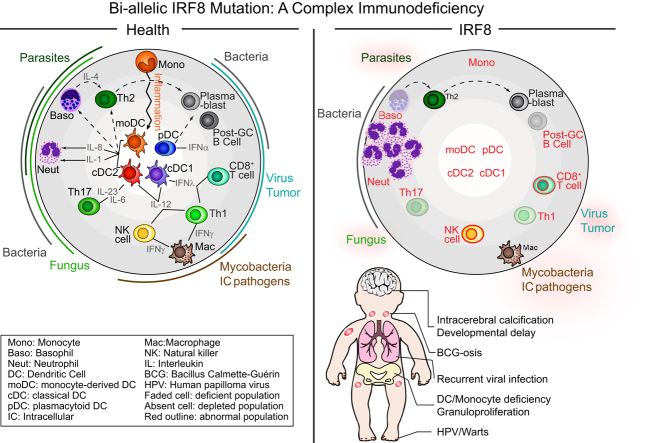

Effective immunity depends on multiple interacting transcription factors (TFs) that govern hematopoietic development and immune cell function. Members of the interferon regulatory factor (IRF) family of TFs have a dual role, interacting with hematopoietic TFs to determine cell fate and with immune signaling molecules to direct cell responses.^1^ IRF4 and IRF8 are structurally related family members with an N-terminal DNA-binding domain and C-terminal interferon regulatory factor–associated domain (IAD).^2^ They activate or repress transcription at specific DNA motifs in collaboration with other TFs interacting with the IAD.

IRF8 heterodimerization partners include SPI1/PU.1, which binds the Ets–interferon regulatory factor composite element (EICE), frequently activating gene expression. Other IRF family members (IRF1, IRF2, and IRF4) are cofactors at the mostly repressive interferon-stimulated response element (ISRE).^3^, ^4^ IRF8 forms a complex with BATF3 and JUN on the activator protein 1–interferon regulatory factor composite element (AICE) to activate gene expression.^5^ IRF8 plays a critical role in hematopoietic lineage determination through these interactions, governing the development of granulocytes, dendritic cells (DCs), monocytes, and B cells.^6^, ^7^

In myeloid cell development IRF8 is pivotal in determining granulocyte versus monocyte fate. IRF8 competes with CEBPA for binding to chromatin, resulting in attenuation of neutrophil differentiation in granulocyte-monocyte progenitors.^8^ IFR8/PU.1 interaction is critical in monocyte differentiation by marking distal enhancers for subsequent activation by the master regulator Kruppel-like factor 4.^9^ The *Irf8*^−/−^ mouse develops massive expansion of granulocytes and progression to fatal myeloblastic leukemia.^10^ Ly6C^+^ and Ly6C^−^ monocytes are depleted, but some tissue macrophages persist.^11^ The BXH2 mouse is homozygous for the *Irf8*^*R294C*^ mutation in the IAD region of the gene, disrupting association with heterodimerization partners. Monocytes and macrophages are present but rendered hypofunctional in their response to IFN-γ,^12^ production of inflammatory cytokines,^13^ and defense against intracellular infection.^4^, ^14^ These 2 models illustrate the dual hematopoietic and immune response roles of IRF8 and their differential sensitivity to IRF8 deficiency.

DCs are critical for activation of the immune response and arise independently of monocytes. All mammalian species have IFN-α–producing plasmacytoid dendritic cells (pDCs) and 2 myeloid or conventional dendritic cell (cDC) populations.^15^ cDC1s express C-type lectin containing domain type 9A/chemokine XC receptor 1 (CLEC9A/XCR1) and are specialized for cross-presentation, whereas cDC2s express signal regulatory protein α (SIRPA) and mediate T_H_2 and T_H_17 responses. In human subjects they are marked by CD141 and CD1c, respectively.^16^ In mice IRF8 specifies DC lineage in cooperation with Id2.^17^ IRF8 is also upregulated by E2-2^18^ and required for pDC development and function.^19^, ^20^ In cDC1s it interacts with BATF3 to maintain terminal differentiation.^21^ The *Irf8*^−/−^ mouse lacks cDC1s and has reduced numbers of pDCs, whereas selective loss of cDC1s is observed in the hypomorphic BXH2 mouse. cDC2s are preserved in both IRF8-deficient strains through their reciprocal dependence on IRF4.^22^

In B-cell development the balance between IRF8 and IRF4 mirrors that observed in specification of myeloid DCs subsets. IRF8 and IRF4 act redundantly to drive pre–B-cell development but are required differentially to regulate marginal zone versus follicular fate. IRF8 is important in the germinal center reaction and class-switch recombination, whereas IRF4 is necessary for plasma cell differentiation.^23^, ^24^

IRF8 also directly influences the terminal differentiation of natural killer (NK) and T cells. Defects in NK cell maturation and function were reported recently in patients with IRF8 mutations.^25^ Investigation of the *Irf8*^−/−^ mouse has identified defects in T-cell subset differentiation. IRF8 is required for T_H_1 polarization, CD8^+^ effector memory T-cell differentiation,^26^, ^27^ and generation of a T_H_1-associated subset of regulatory T (Treg) cells,^28^ with conflicting reports of the influence of IRF8 on T_H_17 polarization.^29^, ^30^

Human primary immunodeficiencies resulting from single-gene mutations offer unique opportunities to study the development and regulation of immune cells *in vivo*. An infant with impaired immunity to mycobacteria, viruses, and fungi because of a homozygous K108E mutation of IRF8 was described previously.^31^, ^32^ The human phenotype was broadly reminiscent of *Irf8*^−/−^ mice, except that cytopenia encompassed all DC and monocyte subsets. Analysis was complicated by environmental factors of disseminated BCG infection and cytoreductive therapy, and wider cell-specific immune defects were not studied. Here we describe 2 novel alleles of IRF8 in a compound heterozygous patient who was ambulatory between infective exacerbations. This reveals a complex immunodeficiency syndrome comprising profound hematologic defects with widespread immune dysregulation.

## Methods

### Study approval

The study was performed in accordance with the Declaration of Helsinki and approved by the Newcastle and North Tyneside Research Ethics Committee. Written informed consent was obtained from participants before recruitment.

### Whole-exome sequencing

Details of whole-exome sequencing and analysis are described in the Methods section in this article's Online Repository at www.jacionline.org.

### Serum autoantibody analysis across human protein microarray

Sera from the patient and 2 age-matched control subjects were incubated on HuProt human protein microarrays (CDI Laboratories, Baltimore, Md). Details are available in the Methods section in this article's Online Repository.

### Luciferase reporter assays

Expression constructs encoding human PU.1, Spi-B transcription factor (SPIB), BATF, and IRF1 have been described elsewhere.^33^, ^34^ Human *JUNB* and *IRF8* wild-type and mutated cDNA sequences preceded by a hemagglutinin (HA) epitope tag were purchased from GenScript (Piscataway, NJ) and cloned into pIRES2-EGFP expression vectors (Clontech, Mountain View, Calif). Luciferase reporter constructs are described in the Methods section in this article's Online Repository. HeLa cells were seeded at 1 × 10^5^ cells/well in 24-well plates with RPMI plus 10% FBS and transfections were performed in triplicate with GeneJuice Transfection Reagent (Novagen, Merck). Twenty nanograms of expression vector as cotransfected with 150 ng of firefly luciferase vector and 1 ng of pRL-CMV *Renilla* luciferase control. For each condition, the total amount of vector transfected was normalized by inclusion of control pIRES2-EGFP empty vector. Luciferase activity was assayed 24 hours after transfection by using the Promega luciferase dual assay system (Promega, Madison, Wis) and analyzed on a Berthold Lumat LB Luminometer (Berthold Technologies, Bad Wildbad, Germany). For each condition, luciferase activity was expressed relative to the average reading from the empty vector transfections and normalized to 1. Comparable expression of IRF8 wild-type and variant alleles by transected cells was confirmed by means of immunoblotting for the HA epitope tag.

### Electrophoretic mobility shift assays

Nuclear extracts were prepared from HeLa cells transfected with expression vectors for PU.1, SPIB, IRF1, BATF, JUNB, IRF8 WT, IRF8 R83C, or IRF8 R291Q, as previously described.^33^ Double-stranded DNA probes end-labeled with [γ-^32^P] ATP using T4 polynucleotide kinase were incubated with appropriate nuclear extract in the presence of poly(dI:dC; Amersham Biosciences, Buckinghamshire, United Kingdom) for 30 minutes at room temperature. The probes are detailed in the Methods section in this article's Online Repository.

### IRF8 protein analysis

HeLa cells were transfected with IRF8 constructs by using GeneJuice, as described above. Twenty-four hours after transfection, whole-cell lysates were prepared by using RIPA buffer (20 mmol/L Tris-HCl [pH 7.5], 150 mmol/L NaCl, 1 mmol/L EDTA, 1% NP-40, and 1% sodium deoxycholate). Alternatively, subcellular fractions were prepared with NE-PER Nuclear and Cytoplasmic Extraction Reagents (Thermo Fisher Scientific, Waltham, Mass), according to the manufacturer's instructions. Whole-cell equivalents or separated fractions were run on a 10% SDS-PAGE gel, transferred to nitrocellulose, and probed with antibody to HA (Ab9110; Abcam, Cambridge, United Kingdom), IRF8 (sc-13043; Santa Cruz Biotechnology, Dallas, Tex), α-actin (clone AC-15; Sigma, St Louis, Mo), or α-tubulin (DMIA; Millipore, Temecula, Calif) and subsequently detected by using horseradish peroxidase–coupled secondary antibody and ECL reagent (Thermo Fisher Scientific).

### Cell culture and functional assays

PBMCs were prepared by means of density gradient centrifugation, and neutrophils were harvested from the red cell/granulocyte layer, according to standard protocols. Skin from the patient and control subjects undergoing mammoplasty surgery was processed, as previously described.^35^ Dihydrorhodamine oxidative burst was performed with Phagoburst (Glycotope Biotechnology, Berlin, Germany). PHA responses were determined by means of incorporation of tritiated thymidine after 6 days of culture. Whole-blood cytokine assays were performed, as previously described.^36^

Cytokines and chemokines in serum and bronchoalveolar lavage (BAL) fluid supernatants were measured with Luminex by using ProcartaPlex 34-plex Immunoassay (eBioscience, San Diego, Calif) on Qiagen Liquichip 200 (Qiagen, Hilden, Germany) running Luminex 100 integrated system software (version 2.3; Luminex, Austin, Tex). Standard curves were constructed to interpolate analytes by using Procartaplex Analyst (version 1.0). The mean of technical duplicates was recorded.

Intracellular cytokine measurement was performed on sorted T-cell subsets. Fifty thousand cells in 200 μL of RPMI plus 10% FBS were stimulated for 3 hours with 0.02 μg/mL phorbol 12-myristate 13-acetate (Sigma-Aldrich) and 0.5 μg/mL ionomycin (Sigma-Aldrich) at 37°C, followed by 2 μg/mL brefeldin A (Sigma-Aldrich) for a further 3 hours. Cells were washed and stained for intracellular cytokines after permeabilization with FOXP3 staining buffer (eBioscience).

### Flow cytometry and microscopy

PBMCs and skin mononuclear cell preparations or lymphoblastoid cell lines were stained in aliquots of 1 to 3 × 10^6^ cells in 50 μL of Dulbecco-PBS with 2% FCS and 0.4% EDTA. Dead cells, usually less than 5%, were excluded by using 4′-6-diamidino-2-phenylindole dihydrochloride (Partec, Görlitz, Germany). Intracellular IRF8 staining was performed after fixation and permeabilization, according to the manufacturer's instructions (eBioscience). Analysis was performed with an LSRFortessa X-20 and sorting with a FACSAria III (BD Biosciences, San Jose, Calif). Data were processed with FlowJo software (Tree Star, Ashland, Ore). Absolute cell counts were obtained with TruCount tubes (BD Biosciences) with 200 μL of whole blood with 900 μL of lysis buffer. Fluorescence microscopy of epidermal sheets and cytospin and Giemsa staining of PBMCs was performed, as previously described,^36^ with an Axioplan 2 microscope (Carl Zeiss, Oberkochen, Germany) with an EC Plan-Neofluar ×40 NA 0.75 lens. Antibodies are listed in Table E1 in this article's Online Repository at www.jacionline.org.

### Deep sequencing of the B-cell IgH complementarity-determining region 3

Peripheral blood B cells (>60,000) from patients with the 83C/291Q and K108E mutations and 3 age-matched controls were sorted by means of fluorescence-activated cell sorting to greater than 95% purity, and genomic DNA was extracted by using standard methods. High-throughput sequencing of the B-cell receptor heavy chain (IgH) complementarity-determining region 3 (CDR3) was undertaken by using the Adaptive Biotechnologies immunoSEQ Assay (www.adaptivebiotech.com). Further details are available in the Methods section in this article's Online Repository.

### Statistical analysis

Graphs were plotted with Prism software (version 5; GraphPad Software, La Jolla, Calif) and means, SDs, *z* scores, and Student *t* test results were calculated within this software. For gene ontology analysis and B-cell receptor IgH gene use, R version 3.3.0 was used, together with the bimaRt, GOstats, and ggplot2 packages.

## Results

### Immunodeficiency

**Fig 1:**
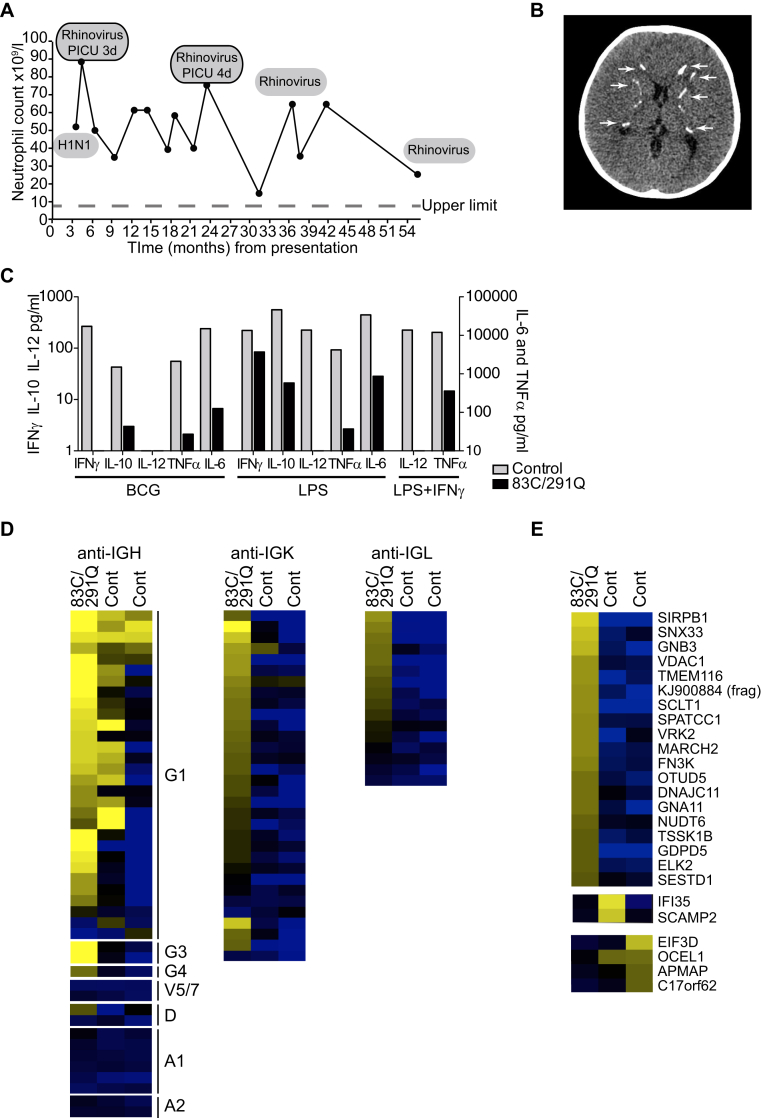
Immunodeficiency. **A,** Clinical course of the patient showing peripheral blood *(PB)* neutrophil counts (× 10^9^/L) compared with the upper limit of normal. *Black dots* represent hospital admissions, *boxes* indicate pathogens isolated, and *outlined boxes* indicate admission to intensive care for respiratory support and length of stay. *PICU*, Pediatric intensive care unit. **B,** Standard noncontrast enhanced computed tomogram showing multifocal bilateral parenchymal calcification *(white arrows)* involving the subcortical white matter, globus pallidus, internal capsule, and dentate nuclei. **C,** Whole-blood cytokine production from the patient bearing the *IRF8^R83C/R291Q^* mutation (black) compared with a travel control subject (gray). **D** and **E,** Antibody reactivity in serum from the patient and 2 age-matched control subjects against a panel of protein targets representing greater than 15,000 human genes (HuProt). Fig 1, *D*, Anti-immunoglobulin heavy chain (IGH) or light chain (IGK and IGL) reactivity after quantile normalization across the 3 samples. Fig 1, *E*, Nonimmunoglobulin antigen reactivity present discretely in the patient or control subjects.

A white male subject born to nonconsanguineous parents had an early history of recurrent severe viral respiratory tract infection with influenza H1N1, rhinovirus, and mycoplasma, often requiring hospitalization and ventilatory support (Fig 1, *A*, and see the Methods section in this article's Online Repository for clinical details). Developmental delay had been noted, and multifocal bilateral calcification was present at age 3 years (Fig 1, *B*). Total CD4^+^ cell, CD8^+^ T-cell, B-cells, and NK cell counts were normal, and T-cell PHA responses were intact (see Table E2 and Fig E4, *B*, in this article's Online Repository at www.jacionline.org). IgA deficiency was noted during infancy (see Table E3 in this article's Online Repository at www.jacionline.org). Whole-blood cytokine assay revealed absent IL-12 production with reduced IFN-γ, IL-6, IL-10, and TNF-α levels in response to BCG, LPS, or LPS and IFN-γ (Fig 1, *C*). No antinuclear antibodies were identified in serum by using BioPlex 2200 screening, but analysis across a human protein microarray (HuProt), representing more than 15,000 genes (see Table E4 in this article's Online Repository at www.jacionline.org), revealed a striking reactivity against immunoglobulin heavy and kappa and lambda light chains compared with 2 age-matched control subjects (Fig 1, *D*). A number of discrete non–immunoglobulin-targeted antibodies were identified in the patient, and a smaller number were identified in control subjects (Fig 1, *E*).

### Compound heterozygous *IRF8* mutations: R83C and R291Q

**Fig 2:**
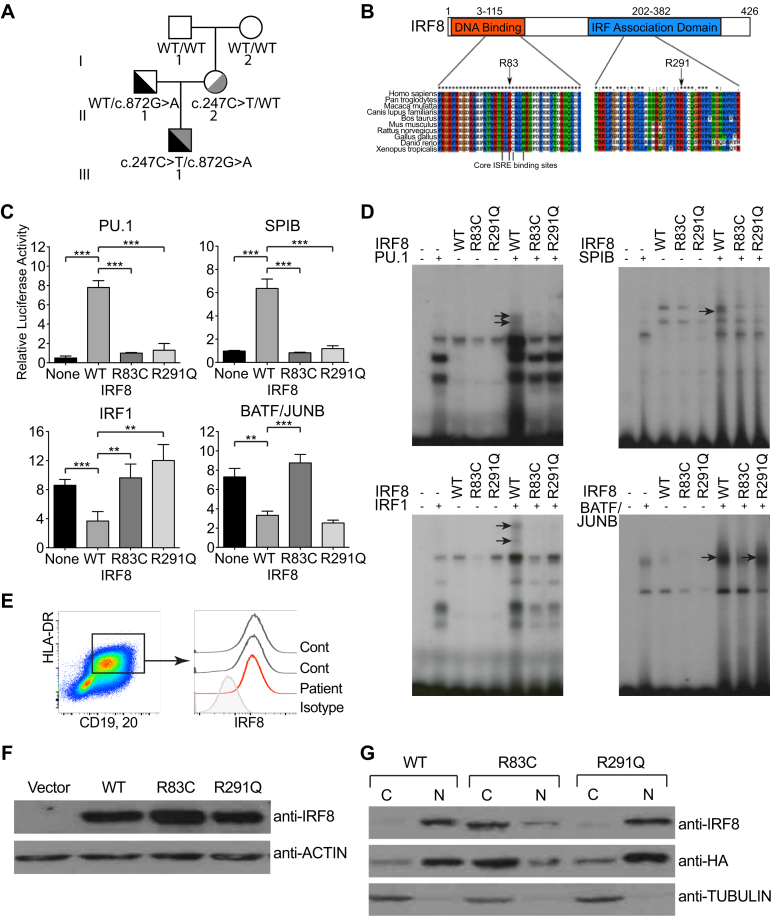
Compound heterozygous *IRF8* mutations R83C and R291Q. **A,***IRF8* genotype of the proband (III.1) and family. **B,** Protein schematic and multiple sequence alignment of *IRF8* orthologs. Mutated amino acids and core ISRE binding residues are marked. **C,** Evaluation of IRF8/cofactor transcriptional potential, as determined by relative luciferase activity of IRF response elements in the absence of IRF8 *(None)* or transactivated by wild-type IRF8 or the variants R83C and R291Q in the presence of PU.1 and *PSMB8* derivative (EICE), SPIB and *PSMB8* derivative (EICE), IRF1 and *TAPASIN* derivative (ISRE), and BATF/JUNB and *IL10* derivative (AICE), as indicated. Graphs show results from 3 experiments. Luciferase activity is displayed relative to cells cotransfected with empty vector normalized to 1. Data were analyzed by using an unpaired *t* test as follows: **P < .01 and ***P < .001. **D,** Evaluation of IRF8/cofactor complex–forming potential, as determined by using EMSAs with nuclear extracts prepared from HeLa cells transfected with *IRF8* WT or the variants R83C and R291Q and mixed with extracts containing PU.1 and *PSMB8* derivative (EICE), SPIB and *PSMB8* (EICE), IRF1 and *TAPASIN* (ISRE), BATF/JUNB and *IL10* (AICE), as indicated. *Arrows* indicate IRF8/cofactor complexes. **E,** Flow cytometric evaluation of intracellular IRF8 protein expression in EBV-transformed B cells from the patient and 2 control subjects. **F,** IRF8 expression in whole-cell lysates from HeLa cells transfected with the indicated constructs. **G,** IRF8 and tubulin expression in cytoplasmic *(C)* and nuclear *(N)* fractions of HeLa cells transfected with the indicated constructs. Transfected IRF8 was detected by using both anti-IRF8 and anti-HA.

Whole-exome sequencing of the patient and both parents identified 2 novel missense mutations in coding regions of *IRF8:* c.247C>T; p.Arg83Cys and c.872G>A; p.Arg291Gln (Fig 2, *A*). Four genes with biallelic variants were identified. Of these, *IRF8* had the highest combined annotation-dependent depletion scores and was most biologically plausible (see Table E5 in this article's Online Repository at www.jacionline.org). Both *IRF8* mutations were absent from ExAC, dbSNP147, EVS, the NHLBI GO Exome sequencing project, and gnomAD databases and 116 locally sequenced samples. Mutated residues were highly conserved between IRF family members and *IRF8* orthologs (Fig 2, *B*).

In luciferase reporter assays neither R83C nor R291Q activated a derivative of the *PSMB8* promoter EICE in the presence of PU.1 or SPIB, and both were unable to repress IRF1-mediated expression through the *TAPASIN* promoter ISRE (Fig 2, *C*). However, a difference was observed in reporter constructs containing the *IL10* AICE activated by BATF/JUNB. This element is repressed by wild-type IRF8. Here the R83C variant was inactive, but R291Q was comparable with wild-type IRF8. These results were consistent with binding patterns seen in electrophoretic mobility shift assays (EMSAs), where only wild-type IRF8 bound to DNA in the presence of the appropriate cofactor, except for the AICE probe, which retained R291Q binding in the presence of BATF/JUNB (Fig 2, *D*). Total IRF8 protein expression was normal in patients' lymphoblastoid cell lines and whole-cell lysates of transfected HeLa cells (Fig 2, *E* and *F*), but in cellular fractionation experiments R83C was unable to translocate to the nucleus (Fig 2, *G*). R291Q showed a nuclear-cytoplasmic distribution similar to wild-type protein.

### Monocyte and DC deficiency with preserved tissue macrophages

**Fig 3:**
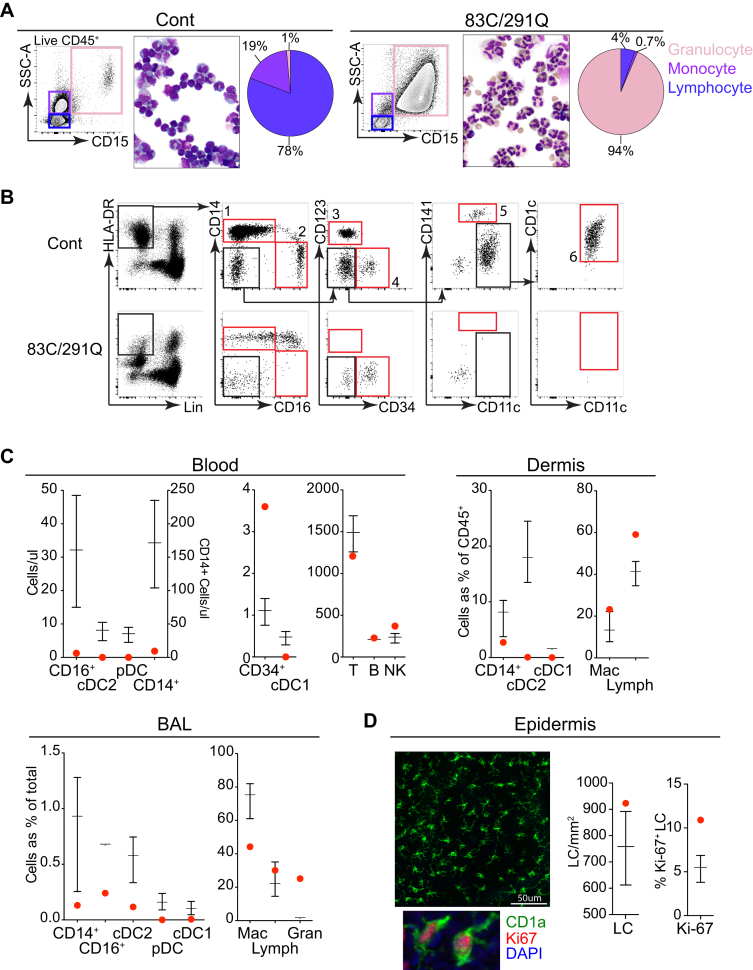
Monocyte and DC deficiency with preservation of macrophages and Langerhans cells *(LCs)*. **A,** Flow cytometric analysis of PBMCs showing CD15^−^SSC^low^ lymphocytes, CD15^−^SSC^med^ monocytes, and residual CD15^+^ granulocytes with morphology confirmed by means of Giemsa staining. Pie charts represent proportions of cells in PBMCs of the patient (83C/291Q mutation) and the mean of control subjects (n = 9). **B,** Flow cytometric PBMC profiling of the patient and a control subject after staining with anti-CD15 to exclude abundant hypogranular neutrophils with high nonspecific antibody binding. The Lineage (CD3, CD19, CD20, and CD56)^−^HLA-DR^+^ gate contains CD14^+^ classical monocytes (gate 1), CD14^−^CD16^+^ nonclassical monocytes (gate 2), CD123^+^ pDCs (gate 3), CD34^+^ progenitors (gate 4), CD141^+^ cDC1s (gate 5), and CD11c^+^CD1c^+^ cDC2s (gate 6). **C,** Flow cytometric profiling of blood, dermis, and BAL fluid. Absolute counts (BD TruCount) in whole blood are shown. Lymphocytes were gated as CD3^+^ T cells, CD19^+^ B cells, and CD3^−^CD56^+^ NK cells. *Bars* represent means ± SDs of 18 control subjects. *Dermis bars* represent means ± ranges of 3 healthy control subjects. *BAL bars* represent means ± ranges of 4 healthy control subjects. *Gran*, Granulocytes; *Lymph*, lymphocytes; *Mac*, macrophages. **D,** Enumeration of LCs by means of immunofluorescence microscopy of an epidermal sheet from the patient stained with anti-CD1a, anti–Ki-67, and 4′-6-diamidino-2-phenylindole dihydrochloride *(DAPI)*. Numbers of LCs and Ki-67^+^ proportions derived from the mean of 6 fields of view (at ×20 or ×40 magnification) for the patient and 13 or 3 healthy control subjects, respectively. *Bars* represent means ± SDs.

Accurate monocyte and DC profiling was possible only after exclusion of abundant CD15^+^ granulocytes in the PBMC fraction (Fig 3, *A*). This revealed a profound depletion of CD14^+^ classical monocytes, CD16^+^ nonclassical monocytes, CD123^+^ pDCs, CD141^+^ cDC1s, and CD1c^+^ cDC2s (Fig 3, *A-C*, and see Fig E1 in this article's Online Repository at www.jacionline.org). As recently reported, there was a relative excess of CD56^bright^ immature NK cells and depletion of CD56^dim^ mature NK cells (see Fig E1, *D*). In the dermis CD14^+^ monocytes-macrophages, CD141^+^ cDC1s, and CD1c^+^ cDC2s were similarly decreased, whereas lymphocytes and autofluorescent dermal resident macrophages were preserved. Elective BAL fluid was also depleted of DCs and CD14^+^ and CD16^+^ monocyte-derived cells, but side-scatter (SSC)^hi^CD45^hi^CD206^+^ alveolar macrophages and SSC^low^HLA-DR^−^ lymphocytes remained in normal numbers. BAL fluid CD15^+^ granulocytes were approximately 10-fold more abundant than in control subjects (Fig 3, *C*). Proliferating epidermal Langerhans cells were also intact (Fig 3, *D*). BAL fluid cytokine levels were also grossly disturbed (see Fig E2, *A*, in this article's Online Repository at www.jacionline.org).

### Dysregulation of granulopoiesis

**Fig 4:**
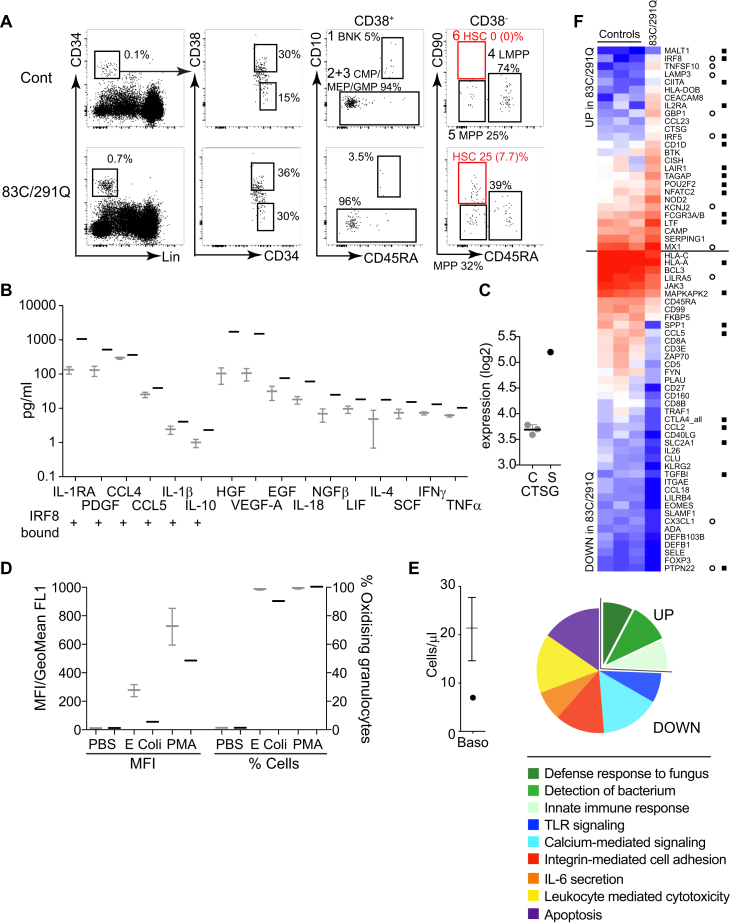
Dysregulated granulopoiesis. **A,** Flow cytometric analysis of CD34^+^ progenitors in PBMCs from a control subject and patient (83C/291Q). Lineage^−^CD34^+^ cells contain CD38^+^CD10^+^ BNK precursors (gate 1), CD10^−^CD45RA^−^ common myeloid progenitor/megakaryocyte-erythroid progenitor (*CMP/MEP*; gate 2), and CD45RA^+^ granulocyte-macrophage progenitor (*GMP*; gate 3). CD38^−^ cells contain CD45RA^+^ lymphoid-primed multipotent progenitors (*LMPP*; gate 4), CD45RA^−^CD90^−^ multipotent progenitors (*MPP*; gate 5), and CD90^+^ hematopoietic stem cells (*HSC*; gate 6). Numbers represent the percentage of cells in the upstream gate or the percentage of CD34^+^ cells. **B,** Serum cytokine analysis with Luminex in a patient with the 83C/291Q mutation (black) compared with 10 control subjects (gray). The graph shows cytokines outside the reference range (*z* score ≥ 2). *+*, Cytokine genes with an IRF8 binding site within 20 kb of the transcription start site. **C,** Cathepsin G *(CTSG)* expression in neutrophils from a patient with the 83C/291Q mutation and 3 control subjects analyzed by using the NanoString Human Immunology V2 panel (control subjects are in gray and the patient with the 83C/291Q mutation is in black). **D,** Dihydrorhodamine oxidative burst response of whole-blood neutrophils to PBS, *Escherichia coli*, and phorbol 12-myristate 13-acetate *(PMA)*. The patient with the 83C/291Q mutation is shown in black. *Gray bars* indicate means ± SDs of 45 control subjects. *MFI*, Mean fluorescence intensity. **E,** Basophils *(Baso)* identified as Lineage^−^HLA-DR^−^CD45^low^CD123^+^ cells. Absolute count (BD TruCount) in whole blood is shown. *Bars* represent means ± SDs of 18 control subjects. **F,** Heat map showing differential gene expression of 1.5 log_2_ or greater from control mean and *z* score of 2 or greater between the patient with the 83C/291Q mutation and control subjects (n = 3). *Open circles*, Genes differentially regulated by interferon; *solid squares*, genes bound by IRF8. Pie charts show gene ontology *(GO)* terms significantly (*P* < .01) enriched after hypergeometric testing of differentially regulated transcripts by using the nCounter Human Immunology V2 panel as the gene universe.

Myeloproliferation consisted of abundant hypogranular neutrophils with reduced density, low light SSC properties, and reduced mRNA expression of a number of primary and secondary granule components (Fig 3, *A*, and see Fig E2, *B*). CD34^+^ progenitors, including CD38^lo^CD45RA^−^CD90^+^ hematopoietic stem cells, were consistently mobilized into the peripheral blood (Fig 4, *A*). Myelopoietins were increased in serum, including the products of several IRF8-bound genes (*IL1RA*, *PDGFB*, *CCL4*, *CCL5*, *IL1B*, and *IL10*), and expression of cathepsin G *(CTSG)* was increased (Fig 4, *B* and *C*). The neutrophil oxidative respiratory burst, tested in the absence of corticosteroid therapy, was reduced in frequency in response to *Escherichia coli* and reduced in intensity (mean fluorescence intensity) in response to both *E coli* and phorbol 12-myristate 13-acetate (Fig 4, *D*). The patient was also basopenic (Fig 4, *E*). Many transcripts expressed in neutrophils were differentially regulated compared with those in healthy control subjects, including a number of interferon-regulated and IRF8-bound genes (Fig 4, *F*, and see Tables E5 and E6 in this article's Online Repository at www.jacionline.org).

### Dysregulation of lymphoid gene expression

A total of 125 transcripts were differentially regulated in sorted lymphoid cells (73 upregulated and 54 downregulated; see Fig E3, *A*, in this article's Online Repository at www.jacionline.org). A core set of 11 genes varied in expression in all lineages. These included upregulation of the antibacterial protein lactoferrin, *NFIL3*, and *CCR1* and downregulation of *TNFAIP3*, *CCL3*, *CCL4*, *HLA-DRA*, *CXCR4*, *IRF1*, *CDKN1A*, and *DUSP4*. More than half of all differentially expressed genes showed interferon-dependent transcription, and approximately a third (45/125) were IRF8 bound according to homology mapping of ChIP-seq data in the mouse.^4^ Within the NanoString code set, a number of pathways were significantly differentially regulated (see Fig E3, *B*, and Table E6). The NanoString panel contained 82 of the DEGs identified by RNA sequencing in the patient with the K108E mutation. Of these, 36 were differentially expressed by both patients, and 15 were IRF8 bound (see Table E7).

### Impairment of CD8^+^ memory differentiation and T_H_1/T_H_17 polarization

**Fig 5:**
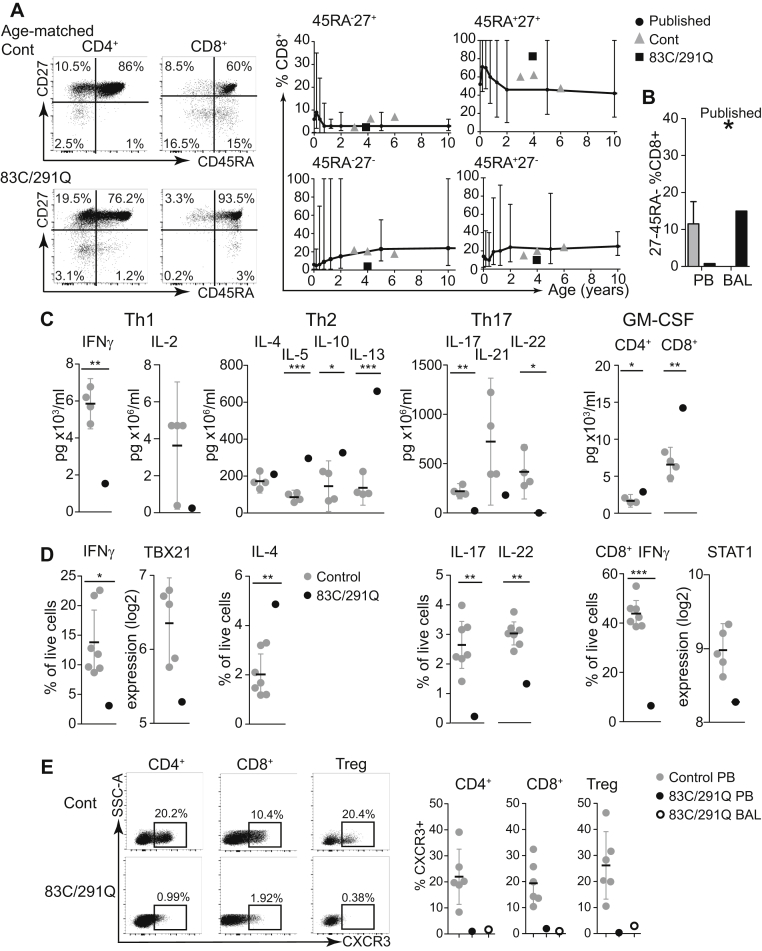
Impairment of T_H_1/T_H_17 and CD8^+^ effector memory T-cell differentiation. **A,** CD4 and CD8 T-cell differentiation defined by expression of CD27 and CD45RA and proportions of each quadrant of CD8^+^ T cells *(outlined gray circle)* relative to 3 age-matched control subjects *(gray triangles)* and published normal ranges *(black bars)*^37^ analyzed as a percentage of total CD8^+^ T cells. **B,** Proportion of CD27^−^CD45RA^−^CD8^+^ T cells in the PB of the patient *(black column)* versus control subjects *(gray column)* and BAL fluid of the patient *(black column)* versus published mean of healthy control subjects *(star)*. **C,** Cytokine secretion into culture medium, as determined by using Luminex technology by purified CD4^+^ and CD8^+^ T cells from the patient and 4 healthy control subjects after phorbol 12-myristate 13-acetate *(PMA)*/ionomycin stimulation. *Bars* represent means ± 95% CIs. **D,** Intracellular cytokine production by purified CD4^+^ and CD8^+^ T cells from the patient and 4 healthy control subjects after PMA/ionomycin stimulation and selected gene expression analysis in the patient with the 83C/291Q mutation (black) versus 5 age-matched control subjects, as determined by using NanoString nCounter technology (Human Immunology V2 panel). *Bars* represent means ± 95% CIs. **E,** Flow cytometric analysis of CXCR3 expression on CD4^+^, CD8^+^, and CD4^+^CD127^−^CD25^+^ Treg cells from a representative control subject and the patient, with the percentage of CXCR3^+^ cells indicated. Summary of CXCR3 expression in blood *(filled circles)* and BAL fluid *(open circles)* CD4^+^, CD8^+^, and CD4^+^CD127^−^CD25^+^ Treg cell subsets from the patient with the 83C/291Q mutation versus 6 healthy control subjects *(gray circles)*. *Bars* represent means ± 95% CIs. *P < .05, **P < .01, and ***P < .001.

T-cell numbers (CD4^+^, CD8^+^, and Treg cells) and PHA responses were normal (see Fig E4, *A* and *B*, in this article's Online Repository at www.jacionline.org). CD4 memory differentiation was normal (see Fig E4, *C*), but CD8^+^CD45RA^−^CD27^-^ effector memory cells were reduced in number in blood and BAL fluid (Fig 5, *A* and *B*,^37^ and see Fig E4, *D*).^38^ T_H_1 and T_H_17 differentiation was impaired and T_H_2 differentiation was increased by cytokine secretion and intracellular staining (Fig 5, *C* and *D*). Production of GM-CSF by CD4^+^ and CD8^+^ T cells was increased (Fig 5, *C*), but CD8^+^ T cells also produced significantly less IFN-γ (Fig 5, *D*). T-bet and signal transducer and activator of transcription 1 (STAT1) expression were also decreased in bulk sorted T cells (Fig 5, *D*). CXCR3 was virtually undetectable (<2% expression) on CD4^+^ T cells, CD8^+^ T cells, and Treg cells isolated from the blood and BAL fluid of the patient (Fig 5, *E*, and see Fig E4, *E*).

### Impaired B-cell maturation and B-cell receptor diversification

**Fig 6:**
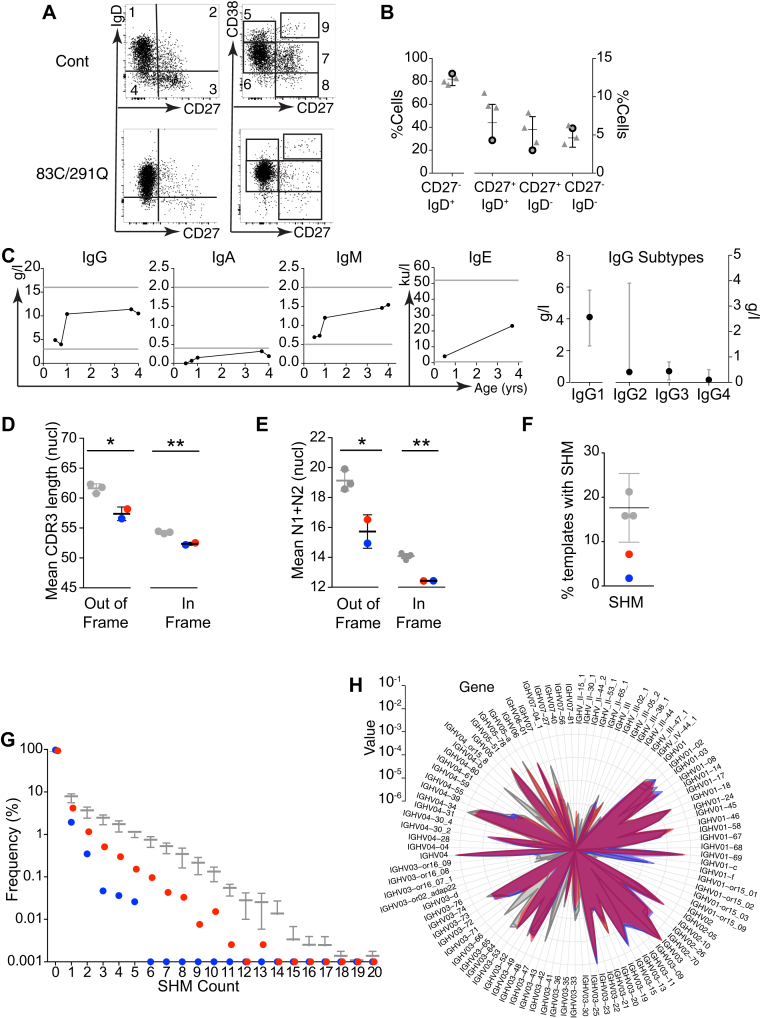
Reduced memory B-cell counts with impaired somatic hypermutation. **A,** Flow cytometric analysis of B-cell phenotype in the patient with 83C/291Q mutations compared with an age-matched control subject. The *left column* shows populations defined by IgD and CD27: naive (gate 1), nonswitched memory (gate 2), switched memory (gate 3), and CD27 memory (gate4). The *right column* shows populations defined by CD38 and CD27 to identify transitional (gate 5), naive mature (gate 6), mature activated (gate 7), memory (gate 8), and plasmablastic (gate 9) cells. **B,** PB B-cell subsets defined by CD27 and IgD expression as a proportion of total B cells from the patient with 83C/291Q mutations *(gray outlined dots)* and 3 local age-matched control subjects *(gray triangles)* plotted against an age-specific (18 months to 4 years) normal range.^39^*Bars* represent means and ranges. **C,** Serum immunoglobulin isotype levels (IgG, IgA, IgM, and IgE) over time. *Horizontal lines* represent upper and lower limits of normal. The *right plot* shows serum levels of IgG subtypes at age 6 months. *Gray bars* show the reference range. **D-H,** B-cell receptor *(BCR)* IgH CDR3 region sequencing of genomic DNA from purified PB B cells (Adaptive Biotechnologies' ImmunoSEQ Assay) from the patient with the 83C/291Q mutations (red), the patient with the K108E mutations (blue), and 3 age-matched control subjects (gray). Fig 6, *D*, Summary of template generation showing the percentage of productive *(Prod)*, out-of-frame *(OoF)*, or stop templates generated. Mean CDR3 length (nucleotides; Fig 6, *E*) and mean number of inserted untemplated nucleotides (N1 and N2; Fig 6, *F*) in out-of-frame and in-frame rearrangements. Fig 6, *G*, Percentage of templates with 1 or more mutated bases. Fig 6, *H*, Frequency of mutated bases per in-frame template expressed as the percentage of total mutations. *Bars* represent means and SDs (Fig 6, *E-H*). Statistics were calculated by using *t* tests as follows: *P < .05 and **P < .01.

Although numbers of CD38^+^CD27^−^ transitional B cells and CD38^hi^CD27^+^ plasmablasts were normal, numbers of both IgD^+^CD27^+^ and IgD^−^CD27^+^ memory cells were reduced (Fig 6, *A* and *B*).^39^ Total IgG, IgM, and IgE levels were normal, but IgA and IgG_2_ levels were reduced (Fig 6, *C*). Although the patient mounted normal specific IgG responses to childhood vaccines, antibodies to HiB and *Pneumococcus* species were not durable and decreased to nonprotective levels by the age of 3 years (see Table E3). Deep sequencing of the immunoglobulin heavy chain (IgH) CDR3 was performed on B-cell DNA isolated from both patients with the R83C/R291Q and those with the K108E/K108E mutations. Clonality and successful template rearrangement were normal (see Fig E5, *A* and *B*, in this article's Online Repository at www.jacionline.org), but CDR3 length was shorter in both productive and nonproductive templates of the patients (Fig 6, *D*, and see Fig E5, *C* and *D*), which is consistent with reduced numbers of nontemplated N1 and N2 nucleotide insertions (Fig 6, *E*). Both patients had proportionally fewer productive templates that had undergone somatic hypermutation (Fig 6, *F*) and a truncated distribution of somatic hypermutation counts with fewer mutated bases per template (Fig 6, *G*). A survey of VH gene expression identified restricted gene use compared with age-matched control subjects (Fig 6, *H*).

## Discussion

This report describes the hematologic defects and immune dysregulation caused by compound heterozygosity of 2 new defective *IRF8* alleles, R83C and R291Q, the latter orthologous to R294C, a hypomorphic mutation responsible for the BXH2 mouse phenotype. Similar to the previously reported child carrying *IRF8*^*K108E/K108E*^, the patient, who was studied in the absence of active infection, had granuloproliferation with profound DC and monocyte deficiency. Further analysis revealed dysregulation of B, NK, and T cells. Although it cannot be proved that lymphoid defects are due to intrinsic loss of IRF8, a number of findings are in accordance with known direct effects of IRF8. These observations shed new light on the mechanisms of immunodeficiency in patients with biallelic *IRF8* mutations, which now include maturation and functional deficits of NK cells; deficiency of T_H_1, T_H_17, and CD8 effector memory responses; and blunted somatic hypermutation, class-switching, and memory B-cell formation.

At the phenotypic level, analysis of this compound heterozygous patient suggests that susceptibility to viral infection, myeloproliferation, and developmental delay associated with cerebral calcification are consistent features of biallelic *IRF8* mutation because they were seen similarly in the patient with homozygous K108E mutation.^31^ The previous case also indicates high susceptibility to mycobacterial infection. Although the compound heterozygous patient was less critically ill, having not been exposed to BCG vaccine in infancy, immunodeficiency was severe enough to warrant hematopoietic stem cell transplantation at age 4 years.

The novel *IRF8* variants, affecting conserved residues, were functionally defective *in vitro*. R83, which is in the DNA-binding domain of the protein, forms hydrogen bonds with the guanine of the GAAA core.^40^, ^41^ R83 belongs to one of the 2 basic residue clusters that form the bipartite nuclear localization signal of IRF8,^42^ explaining the defective nuclear translocation of R83C in cellular localization assays, a feature shared with K108E mutant protein.^32^ R291 is one of only 12 invariant residues in the IADs of IRFs. Crystal structures of IRF3 and IRF5 show that this residue, lying between helix 2 and helix 3, directly interacts with Ets factor and IRF partners in the formation of DNA-binding dimers.^43^ Mutation of the orthologous murine residue 294 in the BXH2 mouse blocks the function of IRF8 in complementation assays and affects the ability of IRF8 to interact with other TFs through the IAD.^44^ Luciferase reporter assays and EMSAs showed that both variants were unable to activate EICE with PU.1 or SPIB and were unable to compete with IRF1 at the ISRE. R83C was unable to bind to BATF/JUN at the AICE but R291Q maintained activity. Although this suggests that R291Q might function effectively as a partner of BATF to facilitate the terminal stages of cDC1 development,^21^ this cannot be interrogated because the failure to coactivate SPI1/PU.1-directed genes blocks DC and monocyte development at an earlier stage. Taken together, the molecular characteristics of the mutations and the phenotypic similarities with the patient carrying null K108E mutations suggest that compound heterozygous R83C and R291Q mutations result in IRF8 activity of less than the threshold for function. Because *IRF8* contains a superenhancer region, gene dose is likely to be critical for normal function.

The mechanism of granuloproliferation in patients with IRF8 deficiency might be multifactorial, with increased production and mobilization but reduced apoptosis of progenitors and neutrophils. First, through cooperation with PU.1 and inhibition of CEBPA at the molecular level, IRF8 is responsible for the balanced generation of myeloid cells in the granulocyte-monocyte progenitor compartment.^45^ Spontaneous granuloproliferation caused by the unopposed action of CEBPA is a consistent finding in both patients with the biallelic *IRF8* mutations, and BXH2 and *Irf8*^−/−^ mouse models.

Second, it was observed that myeloid progenitors, including hematopoietic stem cells, were mobilized into the blood. CXCL12/CXCR4-mediated retention in the bone marrow might have been attenuated because of increased expression of cathepsin G, which degrades CXCL12, and reduced expression of CXCR4, an IRF8 target gene.^46^, ^47^ Levels of growth factors known to mobilize progenitors, including stem cell factor,^48^ hepatocyte growth factor,^49^ and vascular endothelial growth factor,^50^ were also increased.

Finally, *Irf8*^−/−^ progenitors are hyperresponsive to granulocyte colony-stimulating factor^51^ and defective in their inability to upregulate BAX and Fas ligand−mediated apoptosis during termination of emergency myelopoiesis.^52^, ^53^ In keeping with this, apoptosis-related genes were downregulated in the patient's neutrophils.

The expanded neutrophil population was comprised of cells reminiscent of those seen in myelodysplasia; hypogranular with low-light SSC properties,^54^ defects in primary and secondary granules,^55^ and defective respiratory burst responses.^56^ Excessive granulopoiesis appeared to contribute to respiratory complications. At rest, the BAL fluid contained high numbers of granulocytes associated with increased inflammatory cytokine and chemokine levels, including TNF-α, IL-8, CCL2, CCL3, CXCL12, IL-6 and IL-1β. During respiratory tract infection, treatment with glucocorticoids exacerbated the neutrophil response, often leading to a clinical deterioration, whereas treatment with DNAse was effective, which is consistent with lysis of excessive extracellular neutrophil DNA.^57^ In contrast to the neutrophils, basophils were reduced, which is in keeping with a known function of IRF8.^58^

A much broader cytopenia encompassing all monocytes and DCs was seen in both biallelic human subjects compared with the full *Irf8* knockout mouse, which still retains some monocytes and pDCs and has intact cDC2s.^20^ The homozygous R294C BXH2 mouse and heterozygous *Irf8* knockout mouse are only deficient in cDC1s, which require IRF8 for terminal differentiation.^20^ Such selective defects have not yet been observed in human subjects. However, other alleles of *IRF8* have been described that do not impair DC and monocyte development to the same degree. Notably, the heterozygous T80A mutation, although described as dominant negative in reporter assays, had a very modest effect on DC development with apparent loss of cDC2s.^31^ This pattern is unexpected based on mouse models in which cDC2s are always preserved, but the appearance of atypical CD11c^+^CD1c^−^ cells in the human subject suggests that T80A disturbs hematopoiesis by an allele-specific mechanism that has not yet been elucidated. Additional *IRF8* variants recently described by Mace et al^25^ also showed more subtle deficiency in monocytes and DCs but pronounced effects on NK cell development. These variants were localized to the IAD (P224L and A201V). Neither allele was compromised in reporter assays by using the EICE with PU.1 or SPIB or the ISRE with IRF1, suggesting that the loss of protein-protein interactions was responsible for lineage-restricted functional deficits. It is highly likely that other *IRF8* variants will come to light with allele-specific effects on hematopoiesis and immunity. Precise interspecies mapping might not always be possible because of the complexity of interactions between IRF8, IRF4, and other TFs.

Preservation of Langerhans cells and macrophages in states of severe monocytopenia, such as GATA-2 and IRF8 deficiency, remains a key demonstration of the ability of human tissue macrophages to survive without continual replenishment by monocytes.^31^, ^59^ These observations were extended to the alveolar space, which contained large numbers of macrophages. At least partial preservation of macrophages and Langerhans cells is seen in the BXH2 and *Irf8*^−/−^ mouse models,^60^, ^61^ although functional deficits are apparent.^4^, ^12^, ^62^ The contribution of macrophage dysfunction to the human phenotype of IRF8 deficiency remains to be determined, in particular whether the developmental delay and intracerebral calcification seen in patients bearing *IRF8^K108E/K108E^* and *IRF8^R83C/R291Q^* are related to the reported role of Irf8 in murine microglial development and function.^63^, ^64^ There was no evidence for disordered calcium metabolism, but we cannot exclude infectious complications or dysregulated interferon signaling as contributors to this phenotype.

Lymphocyte development and function in the setting of IRF8 deficiency can be altered through both direct cell-intrinsic effects and indirect effects because of defective hematopoiesis and external influences, such as infection. Analysis of the lymphoid compartment showed transcriptomic dysregulation with phenotypic and functional defects in NK, T, and B cells.

Transcriptomic analysis of purified lymphocyte populations revealed enrichment within upregulated genes for response to the pathogen pathways (*LTF*, *CXCL1*, *CD27*, and *TNFRSF*) and immune response (*NFIL3* and *CCR1*), with downregulated genes involved in cytokine signaling (including IFN-γ), cytokine production (*CCL3* and *CCL4*), Toll-like receptor (TLR) signaling (including *TLR3*, *TLR4*, and *TLR5*), cell activation *(HLA-DR)*, and regulation of cell cycle and apoptosis (*DUSP4* and *CDKN1A*). Aberrations in TNF signaling were apparent with upregulation of both TNF and TNF receptor superfamily members but universal downregulation of IRF8-bound TNF-induced *TNFAIP3*, a negative regulator of cytokine-mediated immune and inflammatory responses. *IRF1* expression was also reduced, further contributing to loss of gene expression governed by the IRF8/IRF1 regulome.^4^

Defects in NK cell maturation and function were reported recently as an example of more widespread immune dysregulation caused by *IRF8* mutation by using data from the *IRF8^K108E/K108E^* and *IRF8^R83C/R291Q^* patients. Both show an increased proportion of immature CD56^bright^ NK cells, suggesting impaired NK maturation. Defective NK cell–mediated cytotoxicity was also reported in a patient with mutations in the IAD region (201V/224L) who had severe EBV infection without significant DC or monocyte deficiency.^25^

In the T-cell lineage profound defects in T_H_1, T_H_17, and CD8 effector memory development were observed, together with almost complete absence of CXCR3, the chemokine receptor for CXCL9, CXCL10, and CXCL11, which plays a vital role in the positioning of activated T cells in tissues.^65^ IRF8 is upregulated during T-cell activation,^28^ and experiments with *Irf8*^−/−^ antigen-specific T cells indicate a number of cell-intrinsic regulatory roles for IRF8, including integration of γ-chain cytokine and T-cell receptor signaling pathways, secretion of IFN-γ and cytotoxicity.^26^ IRF8 also regulates STAT1 through mutual promoter binding^66^, ^67^ and represses the GM-CSF gene in T cells.^68^ In *Itgax* (CD11c) conditional *Irf8* knockouts, T-cell dysfunction was proposed to be largely extrinsic through the loss of cDC1, although recombination was also observed in lymphoid cells, which express CD11c when activated.^69^ Evidence for an intrinsic role of IRF8 in T_H_17 differentiation is conflicting.^69^ It is likely that the strength of T-cell receptor signaling is compromised significantly both by the lack of DCs and intrinsic defects including attenuated STAT1-mediated signaling, especially critical for IFN-γ–driven T_H_1 differentiation. Both contribute potentially to the failure of T_H_1, T_H_17, and CD8 effector memory differentiation and lack of CXCR3 expression, leading to compromised peripheral tissue T-cell homeostasis. Transcriptomic analysis of purified T cells showed global downregulation of pathways involved in cell signaling, cytokine production, and cell activation, which is consistent with gene expression analysis of whole PBMCs by RNA sequencing in the *IRF8^K108E/108E^* patient.^32^

In B cells lineage-specific knockout in mice indicates that IRF8 is required at several stages of B-cell development, including maintenance of central tolerance in the bone marrow, differentiation of follicular B cells, germinal center formation, antibody affinity maturation, and memory cell formation.^7^, ^23^ Correlates of these intrinsic effects were observed in humans. In both *IRF8^R83C/R291Q^* and *IRF8^K108E/K108E^* patients, the extent and complexity of somatic hypermutation was reduced, a factor directly associated with serious respiratory infection in patients with common variable immunodeficiency.^70^ Analysis of CDR3 sequences in both patients compared to age-matched control subjects showed shorter lengths with fewer N1 and N2 insertions. Although terminal deoxynucleotidyl transferase, which is responsible for N insertions, is not known to be a direct target of IRF8, impairment is seen in other conditions with perturbed hematopoiesis and aberrant B-cell development.^71^ The restricted VH gene repertoire might also reflect abnormal V(D)J recombination within the bone marrow^72^ or represent defective secondary receptor editing.^73^ Although shorter in-frame CDR3 regions were appropriately selected for compared with out-of-frame sequences, suggesting intact central B-cell tolerance,^74^ the presence of widespread anti-immunoglobulin reactivity across a human proteome microarray was striking in the serum of the patient carrying *IRF8^R83C/R291Q^* compared with age-matched control subjects. The clinical significance of autoreactive antibodies in this age group is uncertain, but in the absence of autoimmune clinical symptoms, the anti-immunoglobulin activity might represent an appropriate response to recurrent infections.^75^ Recent data indicate that IgA deficiency might be explained by a lack of DC–B-cell interaction in Peyer patches.^76^

In summary, this report defines 2 new variants of *IRF8* associated with defective function in a compound heterozygous patient. Profound cytopenia of DCs and monocytes in the context of granuloproliferation was confirmed as a consistent feature of immunodeficiency compounded by intrinsic and extrinsic dysregulation of lymphoid cell development, maturation, and responses. These observations are broadly consistent with the notion that IRF8 controls a set of multilineage functions that hone protective immunity to viral infection and intracellular pathogens. These range from governing the hematopoietic development of cross-presenting cDC1s, interferon-producing pDCs, and monocytes, to the fine-tuning of NK maturation, T_H_1 and T_H_17 cell polarization, effector memory development, and durable production of high-affinity class-switched antibody. This is achieved through multilevel and multilineage control of gene expression, the failure of which is mirrored by a complex syndrome of immunodeficiency.

**Key messages**

• Compound heterozygous IRF8 mutations R83C/R291Q are described in a patient with frequent viral respiratory tract infections.
• Immunodeficiency is due to DC deficiency and monocytopenia with myeloproliferation and widespread immune dysregulation.

## Supplementary data

Online Repository text

## References

[1] Tamura T., Yanai H., Savitsky D., Taniguchi T. (2008). The IRF family transcription factors in immunity and oncogenesis. Annu Rev Immunol.

[2] Glasmacher E., Agrawal S., Chang A.B., Murphy T.L., Zeng W., Vander Lugt B. (2012). A genomic regulatory element that directs assembly and function of immune-specific AP-1-IRF complexes. Science.

[3] Kanno Y., Levi B.Z., Tamura T., Ozato K. (2005). Immune cell-specific amplification of interferon signaling by the IRF-4/8-PU.1 complex. J Interferon Cytokine Res.

[4] Langlais D., Barreiro L.B., Gros P. (2016). The macrophage IRF8/IRF1 regulome is required for protection against infections and is associated with chronic inflammation. J Exp Med.

[5] Murphy T.L., Tussiwand R., Murphy K.M. (2013). Specificity through cooperation: BATF-IRF interactions control immune-regulatory networks. Nat Rev Immunol.

[6] Tamura T., Kurotaki D., Koizumi S. (2015). Regulation of myelopoiesis by the transcription factor IRF8. Int J Hematol.

[7] Shukla V., Lu R. (2014). IRF4 and IRF8: governing the virtues of B Lymphocytes. Front Biol (Beijing).

[8] Kurotaki D., Yamamoto M., Nishiyama A., Uno K., Ban T., Ichino M. (2014). IRF8 inhibits C/EBPalpha activity to restrain mononuclear phagocyte progenitors from differentiating into neutrophils. Nat Commun.

[9] Kurotaki D., Osato N., Nishiyama A., Yamamoto M., Ban T., Sato H. (2013). Essential role of the IRF8-KLF4 transcription factor cascade in murine monocyte differentiation. Blood.

[10] Holtschke T., Lohler J., Kanno Y., Fehr T., Giese N., Rosenbauer F. (1996). Immunodeficiency and chronic myelogenous leukemia-like syndrome in mice with a targeted mutation of the ICSBP gene. Cell.

[11] Hagemeyer N., Kierdorf K., Frenzel K., Xue J., Ringelhan M., Abdullah Z. (2016). Transcriptome-based profiling of yolk sac-derived macrophages reveals a role for Irf8 in macrophage maturation. EMBO J.

[12] Hu X., Ivashkiv L.B. (2009). Cross-regulation of signaling pathways by interferon-gamma: implications for immune responses and autoimmune diseases. Immunity.

[13] Dror N., Alter-Koltunoff M., Azriel A., Amariglio N., Jacob-Hirsch J., Zeligson S. (2007). Identification of IRF-8 and IRF-1 target genes in activated macrophages. Mol Immunol.

[14] Marquis J.F., LaCourse R., Ryan L., North R.J., Gros P. (2009). Disseminated and rapidly fatal tuberculosis in mice bearing a defective allele at IFN regulatory factor 8. J Immunol.

[15] Guilliams M., Ginhoux F., Jakubzick C., Naik S.H., Onai N., Schraml B.U. (2014). Dendritic cells, monocytes and macrophages: a unified nomenclature based on ontogeny. Nat Rev Immunol.

[16] Collin M., McGovern N., Haniffa M. (2013). Human dendritic cell subsets. Immunology.

[17] Jackson J.T., Hu Y., Liu R., Masson F., D’Amico A., Carotta S. (2011). Id2 expression delineates differential checkpoints in the genetic program of CD8alpha+ and CD103+ dendritic cell lineages. EMBO J.

[18] Ghosh H.S., Cisse B., Bunin A., Lewis K.L., Reizis B. (2010). Continuous expression of the transcription factor e2-2 maintains the cell fate of mature plasmacytoid dendritic cells. Immunity.

[19] Schiavoni G., Mattei F., Sestili P., Borghi P., Venditti M., Morse H.C. (2002). ICSBP is essential for the development of mouse type I interferon-producing cells and for the generation and activation of CD8alpha(+) dendritic cells. J Exp Med.

[20] Sichien D., Scott C.L., Martens L., Vanderkerken M., Van Gassen S., Plantinga M. (2016). IRF8 transcription factor controls survival and function of terminally differentiated conventional and plasmacytoid dendritic cells, respectively. Immunity.

[21] Grajales-Reyes G.E., Iwata A., Albring J., Wu X., Tussiwand R., Kc W. (2015). Batf3 maintains autoactivation of Irf8 for commitment of a CD8alpha(+) conventional DC clonogenic progenitor. Nat Immunol.

[22] Schlitzer A., McGovern N., Teo P., Zelante T., Atarashi K., Low D. (2013). IRF4 transcription factor-dependent CD11b+ dendritic cells in human and mouse control mucosal IL-17 cytokine responses. Immunity.

[23] Xu H., Chaudhri V.K., Wu Z., Biliouris K., Dienger-Stambaugh K., Rochman Y. (2015). Regulation of bifurcating B cell trajectories by mutual antagonism between transcription factors IRF4 and IRF8. Nat Immunol.

[24] Wang H., Lee C.H., Qi C., Tailor P., Feng J., Abbasi S. (2008). IRF8 regulates B-cell lineage specification, commitment, and differentiation. Blood.

[25] Mace E.M., Bigley V., Gunesch J.T., Chinn I.K., Angelo L.S., Care M.A. (2017). Biallelic mutations in IRF8 impair human NK cell maturation and function. J Clin Invest.

[26] Miyagawa F., Zhang H., Terunuma A., Ozato K., Tagaya Y., Katz S.I. (2012). Interferon regulatory factor 8 integrates T-cell receptor and cytokine-signaling pathways and drives effector differentiation of CD8 T cells. Proc Natl Acad Sci U S A.

[27] Chaix J., Nish S.A., Lin W.H., Rothman N.J., Ding L., Wherry E.J. (2014). Cutting edge: CXCR4 is critical for CD8+ memory T cell homeostatic self-renewal but not rechallenge self-renewal. J Immunol.

[28] Lee W., Kim H.S., Baek S.Y., Lee G.R. (2016). Transcription factor IRF8 controls Th1-like regulatory T-cell function. Cell Mol Immunol.

[29] Ouyang X., Zhang R., Yang J., Li Q., Qin L., Zhu C. (2011). Transcription factor IRF8 directs a silencing programme for TH17 cell differentiation. Nat Commun.

[30] Newman D.M., Leung P.S., Putoczki T.L., Nutt S.L., Cretney E. (2016). Th17 cell differentiation proceeds independently of IRF8. Immunol Cell Biol.

[31] Hambleton S., Salem S., Bustamante J., Bigley V., Boisson-Dupuis S., Azevedo J. (2011). IRF8 mutations and human dendritic-cell immunodeficiency. N Engl J Med.

[32] Salem S., Langlais D., Lefebvre F., Bourque G., Bigley V., Haniffa M. (2014). Functional characterization of the human dendritic cell immunodeficiency associated with the IRF8(K108E) mutation. Blood.

[33] Tooze R.M., Stephenson S., Doody G.M. (2006). Repression of IFN-gamma induction of class II transactivator: a role for PRDM1/Blimp-1 in regulation of cytokine signaling. J Immunol.

[34] Care M.A., Cocco M., Laye J.P., Barnes N., Huang Y., Wang M. (2014). SPIB and BATF provide alternate determinants of IRF4 occupancy in diffuse large B-cell lymphoma linked to disease heterogeneity. Nucleic Acids Res.

[35] Haniffa M., Ginhoux F., Wang X.N., Bigley V., Abel M., Dimmick I. (2009). Differential rates of replacement of human dermal dendritic cells and macrophages during hematopoietic stem cell transplantation. J Exp Med.

[36] Bigley V., Haniffa M., Doulatov S., Wang X.N., Dickinson R., McGovern N. (2011). The human syndrome of dendritic cell, monocyte, B and NK lymphoid deficiency. J Exp Med.

[37] Schatorje E.J., Gemen E.F., Driessen G.J., Leuvenink J., van Hout R.W., de Vries E. (2012). Paediatric reference values for the peripheral T cell compartment. Scand J Immunol.

[38] Mack D.G., Lanham A.M., Palmer B.E., Maier L.A., Fontenot A.P. (2009). CD27 expression on CD4+ T cells differentiates effector from regulatory T cell subsets in the lung. J Immunol.

[39] Morbach H., Eichhorn E.M., Liese J.G., Girschick H.J. (2010). Reference values for B cell subpopulations from infancy to adulthood. Clin Exp Immunol.

[40] Escalante C.R., Yie J., Thanos D., Aggarwal A.K. (1998). Structure of IRF-1 with bound DNA reveals determinants of interferon regulation. Nature.

[41] Fujii Y., Shimizu T., Kusumoto M., Kyogoku Y., Taniguchi T., Hakoshima T. (1999). Crystal structure of an IRF-DNA complex reveals novel DNA recognition and cooperative binding to a tandem repeat of core sequences. EMBO J.

[42] Lau J.F., Parisien J.P., Horvath C.M. (2000). Interferon regulatory factor subcellular localization is determined by a bipartite nuclear localization signal in the DNA-binding domain and interaction with cytoplasmic retention factors. Proc Natl Acad Sci U S A.

[43] Chen W., Lam S.S., Srinath H., Jiang Z., Correia J.J., Schiffer C.A. (2008). Insights into interferon regulatory factor activation from the crystal structure of dimeric IRF5. Nat Struct Mol Biol.

[44] Turcotte K., Gauthier S., Tuite A., Mullick A., Malo D., Gros P. (2005). A mutation in the Icsbp1 gene causes susceptibility to infection and a chronic myeloid leukemia-like syndrome in BXH-2 mice. J Exp Med.

[45] Tsujimura H., Tamura T., Ozato K. (2003). Cutting edge: IFN consensus sequence binding protein/IFN regulatory factor 8 drives the development of type I IFN-producing plasmacytoid dendritic cells. J Immunol.

[46] Petit I., Szyper-Kravitz M., Nagler A., Lahav M., Peled A., Habler L. (2002). G-CSF induces stem cell mobilization by decreasing bone marrow SDF-1 and up-regulating CXCR4. Nat Immunol.

[47] Cho S.Y., Xu M., Roboz J., Lu M., Mascarenhas J., Hoffman R. (2010). The effect of CXCL12 processing on CD34+ cell migration in myeloproliferative neoplasms. Cancer Res.

[48] To L.B., Bashford J., Durrant S., MacMillan J., Schwarer A.P., Prince H.M. (2003). Successful mobilization of peripheral blood stem cells after addition of ancestim (stem cell factor) in patients who had failed a prior mobilization with filgrastim (granulocyte colony-stimulating factor) alone or with chemotherapy plus filgrastim. Bone Marrow Transplant.

[49] Tajima F., Tsuchiya H., Nishikawa K., Kataoka M., Hisatome I., Shiota G. (2010). Hepatocyte growth factor mobilizes and recruits hematopoietic progenitor cells into liver through a stem cell factor-mediated mechanism. Hepatol Res.

[50] Aicher A., Heeschen C., Mildner-Rihm C., Urbich C., Ihling C., Technau-Ihling K. (2003). Essential role of endothelial nitric oxide synthase for mobilization of stem and progenitor cells. Nat Med.

[51] Scheller M., Foerster J., Heyworth C.M., Waring J.F., Lohler J., Gilmore G.L. (1999). Altered development and cytokine responses of myeloid progenitors in the absence of transcription factor, interferon consensus sequence binding protein. Blood.

[52] Yang J., Hu X., Zimmerman M., Torres C.M., Yang D., Smith S.B. (2011). Cutting edge: IRF8 regulates Bax transcription in vivo in primary myeloid cells. J Immunol.

[53] Hu L., Huang W., Hjort E.E., Bei L., Platanias L.C., Eklund E.A. (2016). The interferon consensus sequence binding protein (Icsbp/Irf8) is required for termination of emergency granulopoiesis. J Biol Chem.

[54] van Lochem E.G., van der Velden V.H., Wind H.K., te Marvelde J.G., Westerdaal N.A., van Dongen J.J. (2004). Immunophenotypic differentiation patterns of normal hematopoiesis in human bone marrow: reference patterns for age-related changes and disease-induced shifts. Cytometry B Clin Cytom.

[55] Bick R.L., Laughlin W.R. (1993). Myelodysplastic syndromes. Lab Med.

[56] Moretti S., Lanza F., Spisani S., Latorraca A., Rigolin G.M., Giuliani A.L. (1994). Neutrophils from patients with myelodyplastic syndromes: relationship between impairment of granular contents, complement receptors, functional activities and disease status. Leuk Lymphoma.

[57] Porto B.N., Stein R.T. (2016). Neutrophil extracellular traps in pulmonary diseases: too much of a good thing. Front Immunol.

[58] Sasaki H., Kurotaki D., Osato N., Sato H., Sasaki I., Koizumi S. (2015). Transcription factor IRF8 plays a critical role in the development of murine basophils and mast cells. Blood.

[59] Bigley V., Collin M. (2011). Dendritic cell, monocyte, B and NK lymphoid deficiency defines the lost lineages of a new GATA-2 dependent myelodysplastic syndrome. Haematologica.

[60] Yamamoto M., Kato T., Hotta C., Nishiyama A., Kurotaki D., Yoshinari M. (2011). Shared and distinct functions of the transcription factors IRF4 and IRF8 in myeloid cell development. PLoS One.

[61] Schiavoni G., Mattei F., Borghi P., Sestili P., Venditti M., Morse H.C. (2004). ICSBP is critically involved in the normal development and trafficking of Langerhans cells and dermal dendritic cells. Blood.

[62] Berghout J., Langlais D., Radovanovic I., Tam M., MacMicking J.D., Stevenson M.M. (2013). Irf8-regulated genomic responses drive pathological inflammation during cerebral malaria. PLoS Pathog.

[63] Kierdorf K., Erny D., Goldmann T., Sander V., Schulz C., Perdiguero E.G. (2013). Microglia emerge from erythromyeloid precursors via Pu.1- and Irf8-dependent pathways. Nat Neurosci.

[64] Masuda T., Tsuda M., Yoshinaga R., Tozaki-Saitoh H., Ozato K., Tamura T. (2012). IRF8 is a critical transcription factor for transforming microglia into a reactive phenotype. Cell Rep.

[65] Groom J.R., Luster A.D. (2011). CXCR3 in T cell function. Exp Cell Res.

[66] Mancino A., Termanini A., Barozzi I., Ghisletti S., Ostuni R., Prosperini E. (2015). A dual cis-regulatory code links IRF8 to constitutive and inducible gene expression in macrophages. Genes Dev.

[67] Kubosaki A., Lindgren G., Tagami M., Simon C., Tomaru Y., Miura H. (2010). The combination of gene perturbation assay and ChIP-chip reveals functional direct target genes for IRF8 in THP-1 cells. Mol Immunol.

[68] Paschall A.V., Zhang R., Qi C.F., Bardhan K., Peng L., Lu G. (2015). IFN regulatory factor 8 represses GM-CSF expression in T cells to affect myeloid cell lineage differentiation. J Immunol.

[69] Luda K.M., Joeris T., Persson E.K., Rivollier A., Demiri M., Sitnik K.M. (2016). IRF8 transcription-factor-dependent classical dendritic cells are essential for intestinal T cell homeostasis. Immunity.

[70] Andersen P., Permin H., Andersen V., Schejbel L., Garred P., Svejgaard A. (2005). Deficiency of somatic hypermutation of the antibody light chain is associated with increased frequency of severe respiratory tract infection in common variable immunodeficiency. Blood.

[71] Roskin K.M., Simchoni N., Liu Y., Lee J.Y., Seo K., Hoh R.A. (2015). IgH sequences in common variable immune deficiency reveal altered B cell development and selection. Sci Transl Med.

[72] Rao S.P., Riggs J.M., Friedman D.F., Scully M.S., LeBien T.W., Silberstein L.E. (1999). Biased VH gene usage in early lineage human B cells: evidence for preferential Ig gene rearrangement in the absence of selection. J Immunol.

[73] Luning Prak E.T., Monestier M., Eisenberg R.A. (2011). B cell receptor editing in tolerance and autoimmunity. Ann N Y Acad Sci.

[74] Pathak S., Ma S., Shukla V., Lu R. (2013). A role for IRF8 in B cell anergy. J Immunol.

[75] Ingegnoli F., Castelli R., Gualtierotti R. (2013). Rheumatoid factors: clinical applications. Dis Markers.

[76] Reboldi A., Arnon T.I., Rodda L.B., Atakilit A., Sheppard D., Cyster J.G. (2016). IgA production requires B cell interaction with subepithelial dendritic cells in Peyer’s patches. Science.

