# Supplementary material for: Biallelic interferon regulatory factor 8 mutation: A complex immunodeficiency syndrome with dendritic cell deficiency, monocytopenia, and immune dysregulation
Source: J Allergy Clin Immunol. 2018 Jun;141(6):2234–48. doi: 10.1016/j.jaci.2017.08.044 (PMC5986711; doi:10.1016/j.jaci.2017.08.044)
Supplement: Online Repository text [file mmc1.docx]

# Supplementary Materials

## Clinical details

A Caucasian male child, born at term, presented with respiratory distress at the age of 7 weeks. He had an elevated total white cell count (59 x 10^9^/l) with neutrophilia (49.5 x 10^9^/l), a high C-reactive protein (122mg/l) and right mid-zone consolidation on chest radiograph. He improved with parenteral antibiotic treatment and influenza A virus (H1N1) was subsequently detected in respiratory secretions. Over the next 3 years he was admitted to hospital on 15 occasions for respiratory distress associated with wheeze and bronchospasm, and required intubation and ventilation 3 times. Rhinovirus was detected in respiratory secretions by PCR on 4 such occasions. With no evidence of sensitization to common aeroallergens and normal sweat test and videofluoroscopy, he was labeled as being severely asthmatic and treated accordingly. Unusually for asthma, however, he was refractory to corticosteroids and his condition rapidly deteriorated after treatment with prednisone more than once. Intermittent high fevers, maculopapular rash, lassitude and limb pain were also observed, sometimes with HHV6 viremia.

There was evidence of pulmonary consolidation on several occasions during acute presentations, but his subsequent CT chest showed full resolution of radiological changes. Examination of bronchoalveolar lavage fluid, at the ages of 1 and 3 years, revealed macrophages, neutrophils and bronchial epithelial cells but no evidence of proteinaceous or lipid deposits, or pathogenic organisms. At 12 months, the patient became unwell with measles-like illness following 1^st^ dose of MMR. He developed a high fever 2-3 days after vaccination, followed by a maculo-papular rash 8-9 days later. He recovered spontaneously but did not receive a further dose of MMR.

Clinical immunological investigations showed persistent neutrophilia, eosinophilia but basopenia, relatively normal hemoglobin and platelets. Intermittent monocytopenia was noted on automated blood counting. Lymphocyte subsets and proliferative T cells responses were normal and serum biochemistry was unremarkable **(Table S1).** There was normal expression of CD18 and CD11 (a, b and c) on neutrophils. IgA deficiency in infancy improved over time. Although there was initially a good response to immunization against S pneumoniae and H. influenza, specific antibodies had declined 2yrs after vaccination **(Table S2)**. Anti-nuclear auto-antibodies were not detected by routine clinical screening. There was no family history of recurrent infections or immunodeficiency.

Despite clinical recovery from respiratory infections his neutrophil count remained massively elevated, prompting further investigations to exclude hematological malignancy. Bone marrow examination showed expanded granulopoiesis, with normal proportions of promyelocytes, myelocytes and neutrophils, normal megakaryocyte and red cell development, no increase in blasts and no evidence of dysplasia. Cytogenetic analysis was normal and there was no evidence of BCR-ABL or JAK2 mutation on molecular testing.

During the course of his investigations delayed neurodevelopmental milestones were noted. He did not sit until age 10 months, walked at 30 months and he had abnormal fine motor skills. By the age of 3½ years he had not talked but had good receptive language and was able to make himself understood by non-verbal means. A CT scan of the head at age of 3 years showed an unusual pattern of intracranial calcification with linear calcification of the internal capsule and white matter of the frontal and parietal lobes. This finding was confirmed by MRI examination.

Informed consent was taken for whole exome sequencing in an attempt to define the cause of this complex immunodeficiency disorder. This revealed compound heterozygous mutations in *IRF8*, as described below.

In light of the severity of clinical symptoms, the theoretical risk of myeloid malignancy and the previous successful treatment of a female infant with *IRF8^K108E/K108E^*, the decision to treat with hematopoietic stem cell transplantation (HSCT) was taken. The patient was transferred to a UK center for children with primary immunodeficiency where, at the age of 4yrs, he underwent a matched unrelated donor cord blood HSCT with treosulfan fludarabine and alemtuzumab with ciclosporine and mycophenolate mofetil and graft versus host disease prophylaxis. Engraftment was uneventful and there was complete reconstitution of all cell subsets without graft versus host disease associated with a dramatic improvement in clinical status, confirming a transplantable immune defect. At the time of writing, the patient remains clinically stable with partial donor chimerism.

## Supplementary Methods

### Genetic analysis

Genomic DNA was isolated from whole venous blood from the subject and parents by standard salt extraction, and enriched for exonic sequences by hybridization with Agilent SureSelect Human All Exon v5 Kit. Paired-end sequencing was performed on an Illumina HiSeq 2500 (100 bp reads) according to the manufacturers’ protocols. Whole-genome alignment to GRCh37 was performed with Novoalign (Novocraft Technologies). Alignments were processed in SAM/BAM formats (^1^ using Picard and the Genome Analysis Toolkit (GATK) ^2,^ ^3^. Indel and single-nucleotide variants were called in gVCF format and combined with 116 other samples using the GenotypeGVCFs of the GATK followed by variant quality score recalibration according to GATK best practices. Recalibrated variants were filtered according to a truth-sensitivity tranche threshold of 99.9%. Variants with a minor allele frequency of ≥ 1% in dbSNP138, NHLBI GO Exome Sequencing Project database, or present in 116 locally sequenced samples were removed. The functional consequences of variation were determined with Ensembl’s Variant Effect Predictor^4^, Sift (<http://sift.jcvi.org>), Polyphen (<http://genetics.bwh.harvard.edu/pph2>) and Condel Consensus Deleteriousness score (<http://bg.upf.edu/fannsdb/>). Variants were ranked according to Combined Annotation Dependent Depletion scores ^5^ (https://cadd.gs.washington.edu/score/).

Under a recessive model of inheritance, whole exome sequencing identified <1% minor allele frequency biallelic variants in four genes: *OR6N1*, *CSPG4, TTN* and *IRF8*, **(Table S3).** *OR6N1* encodes an olfactory receptor ^6^ and *PRPH* encodes a peripheral nerve cytoskeletal protein ^7^. Neither is expressed in hematopoietic or immune cells (human protein atlas; [www.proteinatlas.org](http://www.proteinatlas.org)). *IRF8* was identified as the likely candidate locus because the gene is appropriately expressed, the variants had the highest CADD scores (**Table S3**) and the patient was similar to previously described child with homozygous K108E mutations ^8^.

### Auto-antibody detection on human protein microarray

Sera from the patient and two age-matched controls were incubated on HuProt™ human protein microarrays (CDI Laboratories, Baltimore, MD) at 1:1000 dilution, followed by probing with anti-human IgG(H+L)-Alexa546 (Invitrogen) antibodies. A negative control array was processed in parallel without addition of serum. Arrays were scanned on a LS400 microarray scanner (Tecan), and fluorescence intensities of array spots determined by GenePixPro. Data from the negative control array was subtracted from serum array data, to correct for direct binding of the secondary reagent to immobilised proteins. The corrected fluorescence intensities were log2 transformed and quantile normalIPed for the three serum samples.

### Luciferase reporter constructs

Luciferase reporter constructs containing promoter regions for human *PSMB8* or *TAPASIN* have been described previously^9^. The EICE motif within the *PSMB8* vector was modified by the substitution of the underlined C residues for G’s present in the wild-type sequence, GAGGAACTGAAACCG, for enhanced activation. The AICE motif was derived from an intronic region in the human *IL10* locus (hg19 chr1: 206957777-2069580975) and cloned into the pGL2-Promoter vector (Promega).

### EMSA probe sequences

The probes contained the following sequences with the relevant binding sites underlined (only one strand is shown):

EICE motif (*PSMB8* wild-type) (5’-CGGAGGAGGAAGTGAAAGCGAAAGCCACAGA-3’)

EICE motif (*PSMB8 derivative*) (5’-CGGAGGAGGAACTGAAACCGAAAGCCACAGA-3’)

IRFE motif (*TAPASIN*) (5’-TTTGGAGGAAAGTGAAAGTGAAAGGAGGAAG-3’)

AICE motif (*IL10*) (5’-GGTTGCAGTTTCGTTGTGAGTCAGGCCTGCT-3’)

### Transcriptomic analysis

Freshly isolated peripheral blood B cells, NK cells and T cells were sorted into RLT buffer with 0.1% beta-mercaptoethanol at a concentration of 2,000 cells/µl and analyzed using nCounter® Human Immunology V2 panel. Normalization to positive and negative controls and to housekeeping genes was performed using nSolver™ Analysis Software and log_2_ transformed for subsequent analysis. Genes differentially regulated by interferon were identified from Interferome v2.01 at www.interferome.org ^10^. IRF8-bound genes were identified using ChIP-Seq data from Gene Expression Omnibus (GEO) GSM2060952 ^11^. Conversion from mouse to human orthologs was performed using HomoloGene (www.ncbi.nlm.nih.gov/homologene). IRF8 peaks unambiguously mapped to human orthologs were retained. Mouse data were filtered to all peaks within 20kb of transcription start sites and compared against differentially expressed genes from the subject to identify those with ≥1 IRF8 peak. Significance was calculated using hypergeometric testing and p-values corrected using the Benjamin Hochberg correction (false discovery rate 0.05). Heat maps were generated in Multi Experiment Viewer (MeV 4.8). To determine significantly (p<0.01) enriched Gene Ontology (GO) terms and pathways, a dataset of functional terms associated with each gene was constructed from the Ensembl database using ‘biomaRt’ package for ‘R’. Functional analysis was performed using ‘GOstats’ with the ‘universe’ set to all endogenous probes on the NanoString Immunology V2 panel.

### Deep sequencing of B cell IgH CDR3 region

Peripheral blood B cells (>60,000) from 83C/291Q, K108E and three age-matched controls were FACS sorted to >95% purity and genomic DNA extracted using standard methods. High throughput sequencing of the B cell receptor heavy chain (IgH) CDR3 region was undertaken using Adaptive Biotechnologies® immunoSEQ Assay ([www.adaptivebiotech.com](http://www.adaptivebiotech.com)). PCR bias was controlled using a synthetic immune receptor repertoire ^12^. After read normalization, V, D and J gene segments, non-templated bases and bases that had undergone SHM were identified by comparison with all known V, D and J gene and allele members, according to the standard defined by the International ImMunoGeneTics (IMGT®) collaboration. Sequence- and sample-level analyses were performed using ImmunoSEQ Analyzer. Clonality was calculated within the program to provide a measure (0-1) of the shape of the frequency distribution of all productive clones in the sample (inverse of the normalized version of Shannon’s entropy4). No sample had a clone with frequency >0.036%. V region usage was displayed using the ggplot2 R package.

## Supplementary Figures and Tables

### Figure E1: Gating strategy for monocyte DC and NK analysis

(A) Gating strategy for Trucount analysis. CD3^+^ cells (Gate i) contain CD4^+^ (Gate 2) and CD8^+^ (Gate 3) T cell subsets. CD3-CD45lowCD34^+^ progenitors are identified (Gate 1) and the remaining CD3^-^ cells are defined by expression of HLA-DR and CD4, including HLA-DR^+^CD4^-^ (Gate ii) CD19^+^ B cells (Gate 4), HLA-DR-CD4-CD123^+^ basophils (Gate 5) and CD123-CD45bri NK cells which can be further defined as CD16-CD56^bright^ (Gate 6) or CD56^dim^ (Gate 7). HLA-DR^+^CD4^+^ gate (iv) contains CD14^+^ classical (Gate 8) and CD16^+^ (Gate 9) non-classical monocytes, CD123^+^ pDCs (Gate 10), CD141^+^ cDC1 (Gate 11) and CD11c^+^CD1c^+^ cDC2 (Gate 12).

(B) Flow cytometric analysis of dermis from subject and a representative control after collagenase digestion to single cell suspension. The CD45^+^ leukocyte population contained HLA-DR^-^ (SSC^low^) lymphocytes (Gate 1), HLA-DR^+^ auto-fluorescent (AF^+^) macrophages (Gate 2), and in the HLA-DR^+^AF^-^ gate, CD14^+^ monocyte-derived cells (Gate 3), CD14^-^CD141^+^ cDC1 (Gate 4) and CD14^-^CD11c^+^CD1c^+^ cDC2 (Gate 5).

(C) Flow cytometric analysis of leukocytes from density centrifugation of broncho-alveolar lavage (BAL) fluid. The CD45^+^SSC^low^ gate contained HLA-DR^-^AF^-^ or Lin^+^ lymphocytes (Gate 1) and HLA-DR^+^ antigen presenting cells including CD14^+^ (Gate 2) and CD16^+^ (Gate 3) monocyte-derived cells, CD123^+^ pDC (Gate 4), CD141^+^ or BTLA^+^ cDC1 (Gate 5) and CD1c^+^ cDC2 (Gate 6). The CD45^+^SSC^hi^ gate contained HLA-DR^+^CD206^+^ alveolar macrophages (Gate 7) and CD15^+^CD45^low^ granulocytes (Gate 8).

(D) NK cell (CD45^+^HLA-DR^-^CD4^-^CD3^-^CD19^-^) phenotype by flow cytometric analysis in subject and a healthy control and percentage of CD56^bright^ and CD56^dim^ out of total NK cells in subject compared to n=20 controls.


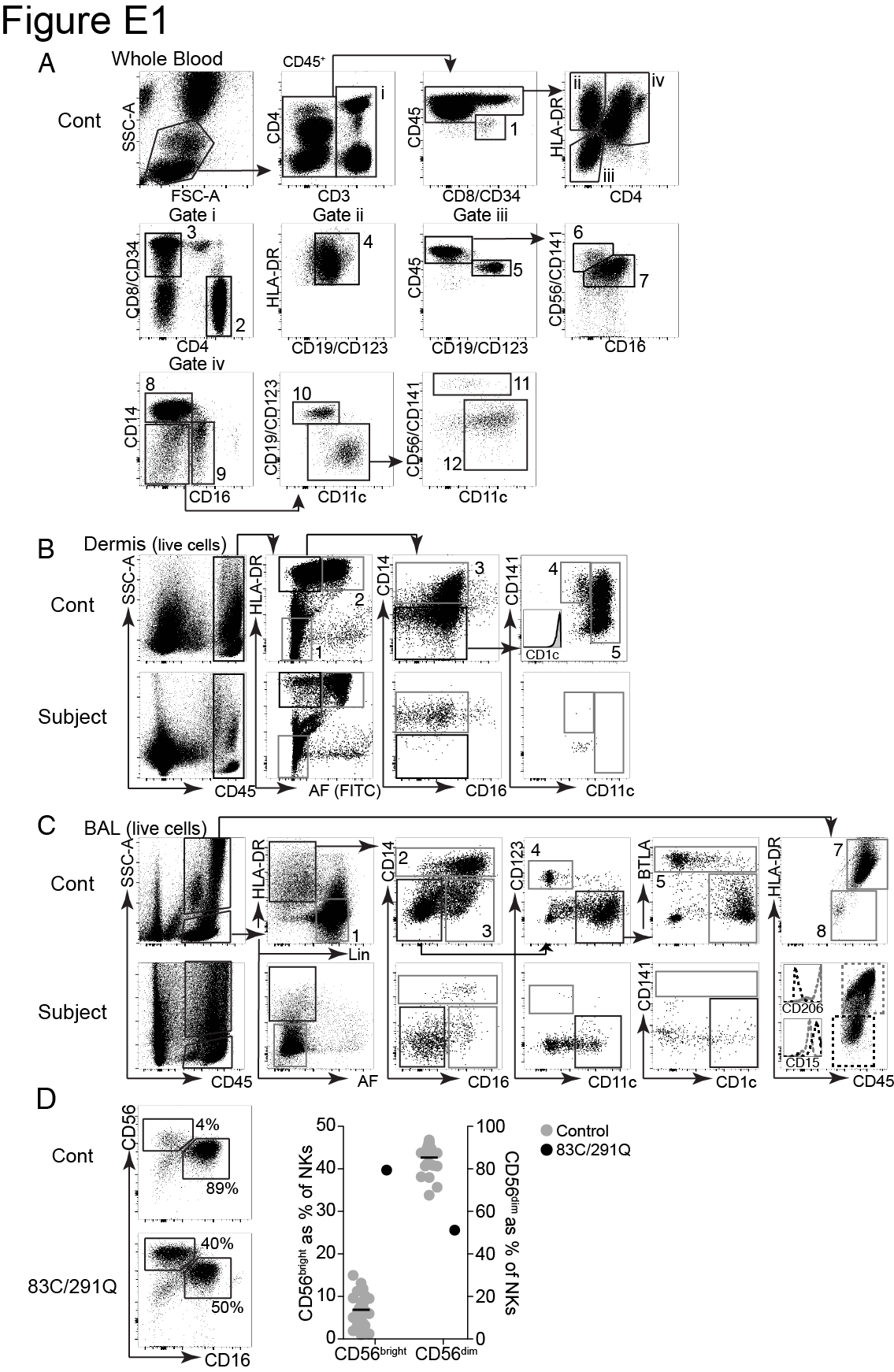


### Figure E2: Cytokine analysis of bronchoalveolar lavage fluid and gene expression of neutrophil granule components

(A) Cytokine and chemokine profile of bronchoalveolar fluid measured by Luminex™ (human cytokine multiplex panels) in subject compared to n=4 healthy controls, expressed as pg/ml: IFNα concentration, IL-27, inflammatory cytokines, cytokines involved in homeostasis of neutrophils in tissue.

(B) Gene expression analysis of granule components in neutrophils of the subject compared to n=3 healthy controls.


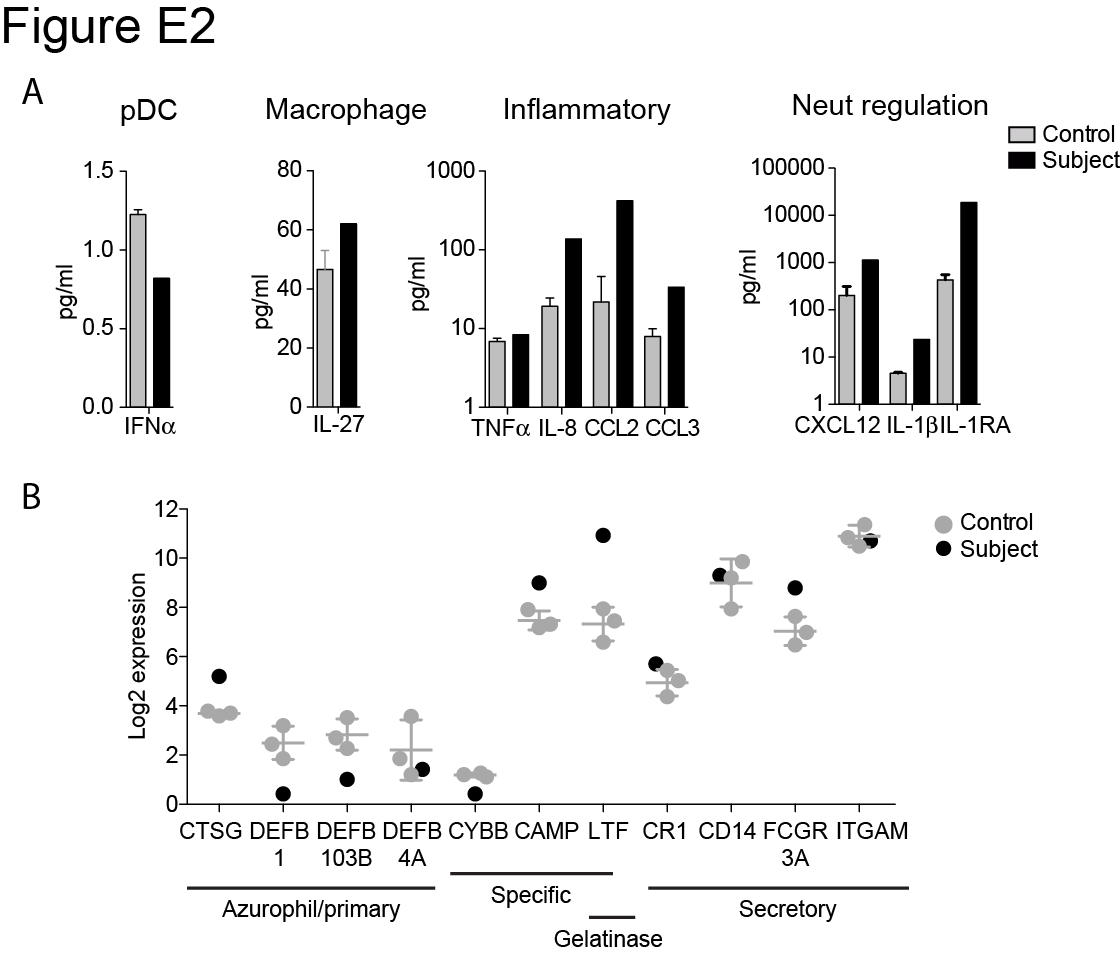


### Figure S3: Gene Expression and ChIP-Seq Analysis

(A) Heat maps showing differential gene expression in FACS sorted B, T and NK cells ≥1.5 log_2_ from control mean and z score ≥2 between 83C/291Q and controls (n=5). Open circles: genes differentially regulated by interferon; filled squares: genes bound by IRF8.

(B) Pie charts: Gene ontology (GO) terms significantly (p<0.01) enriched after hyper-geometric testing of  differentially regulated transcripts using the nCounter Human Immunology V2 panel as the gene universe. Abbreviations: LM, leukocyte migration; CS, complement signaling; A, apoptosis; TM, T cell migration; CC, cell cycle regulation; TNF, tumor necrosis factor; IFN, interferon; TLR, toll-like receptor.


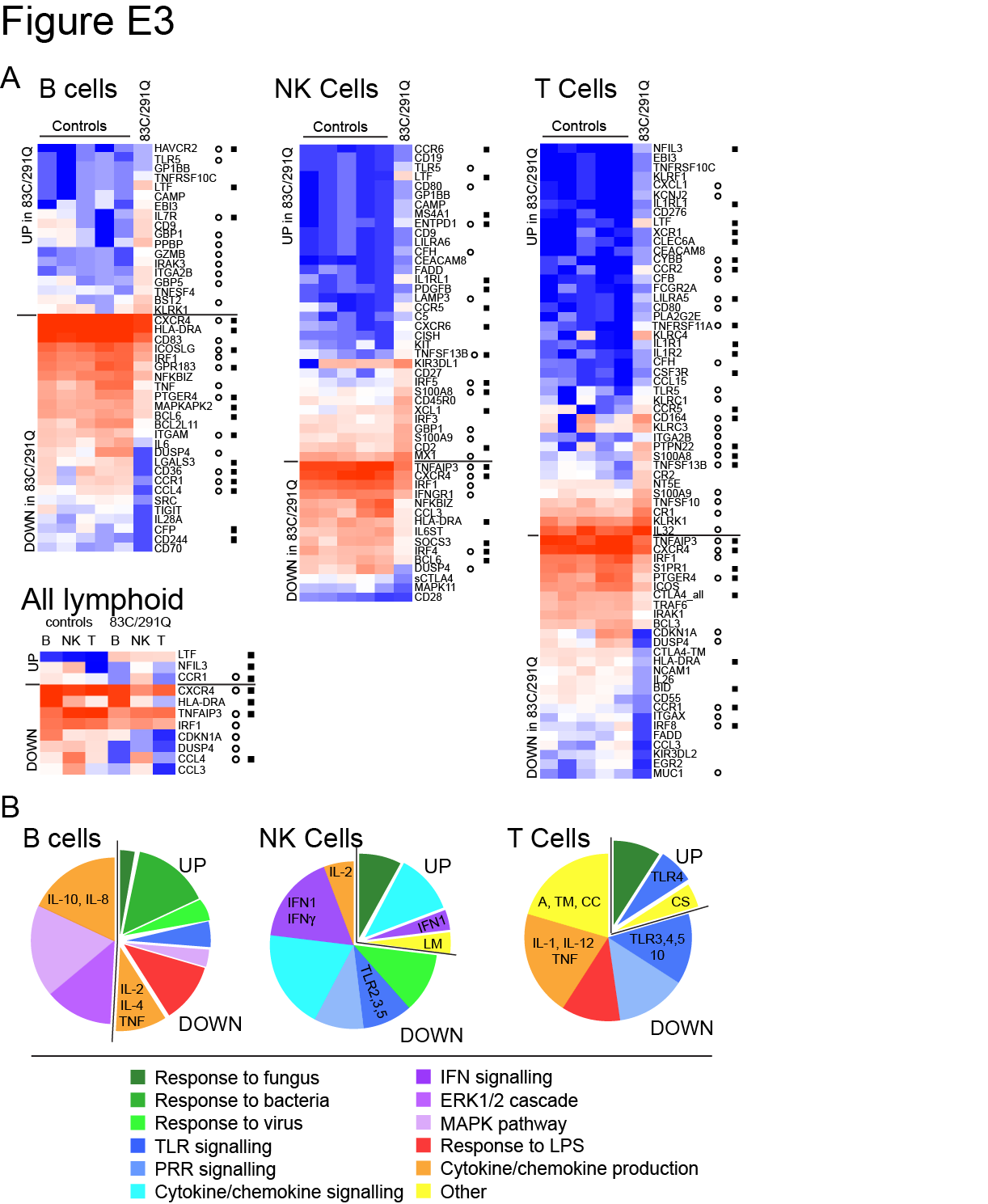


### Figure E4: Additional T cell data

(A) Representative flow cytometry and proportion of CD4^+^ and CD8^+^ T cells in the patient (gray circle) compared with 3 age-matched controls (gray triangles) and published normal ranges (black bars) ^13^.

(B) ^3^H (tritiated) thymidine incorporation by lymphocytes isolated from the patient (black bars) and a control (gray bars) in response to phytohemagglutinin (PHA).

(C) CD4+ T cell memory differentiation assessed by CD45RA and CD27 expression

(D) T cell subsets in BAL of the patient compared with control assessed by CD45RA and CCR7 expression. CD27 expression was not measured for control BAL samples so the patient was also assessed with CD45RA and CCR7 for comparison.

(E) CD27 expression within the CD45RA-CCR7- subset of CD8+ BAL T cells was assessed for comparison with published normal ranges (Figure 5B)


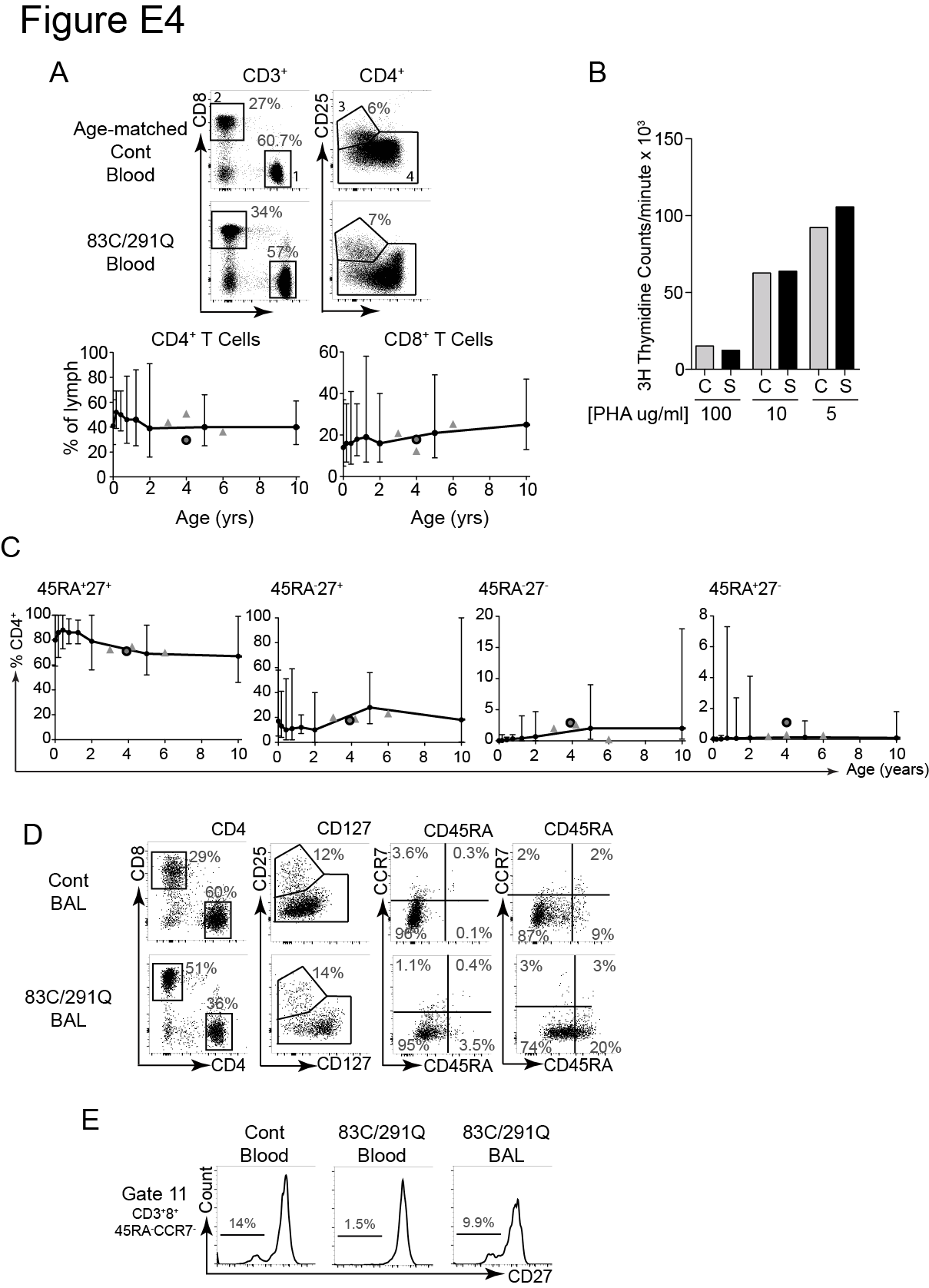


### Figure E5: Additional B cell data

(A) Clonality of B cells from 3 controls and the two subjects. (A,B,D) age-matched controls C1-3 (gray) and patients with *IRF8^R83C/R291Q^* (red) and *IRF8^K108E/K108E^* (blue).

(B) Summary of template generation showing the percentage of productive (Prod), out of frame (OoF) or Stop templates generated in 3 controls and 2 subjects.

(C) In-frame CDR3 template length expressed as % of the total productive template

(D) Out-of-frame CDR3 template length expressed as % of total templates.

**
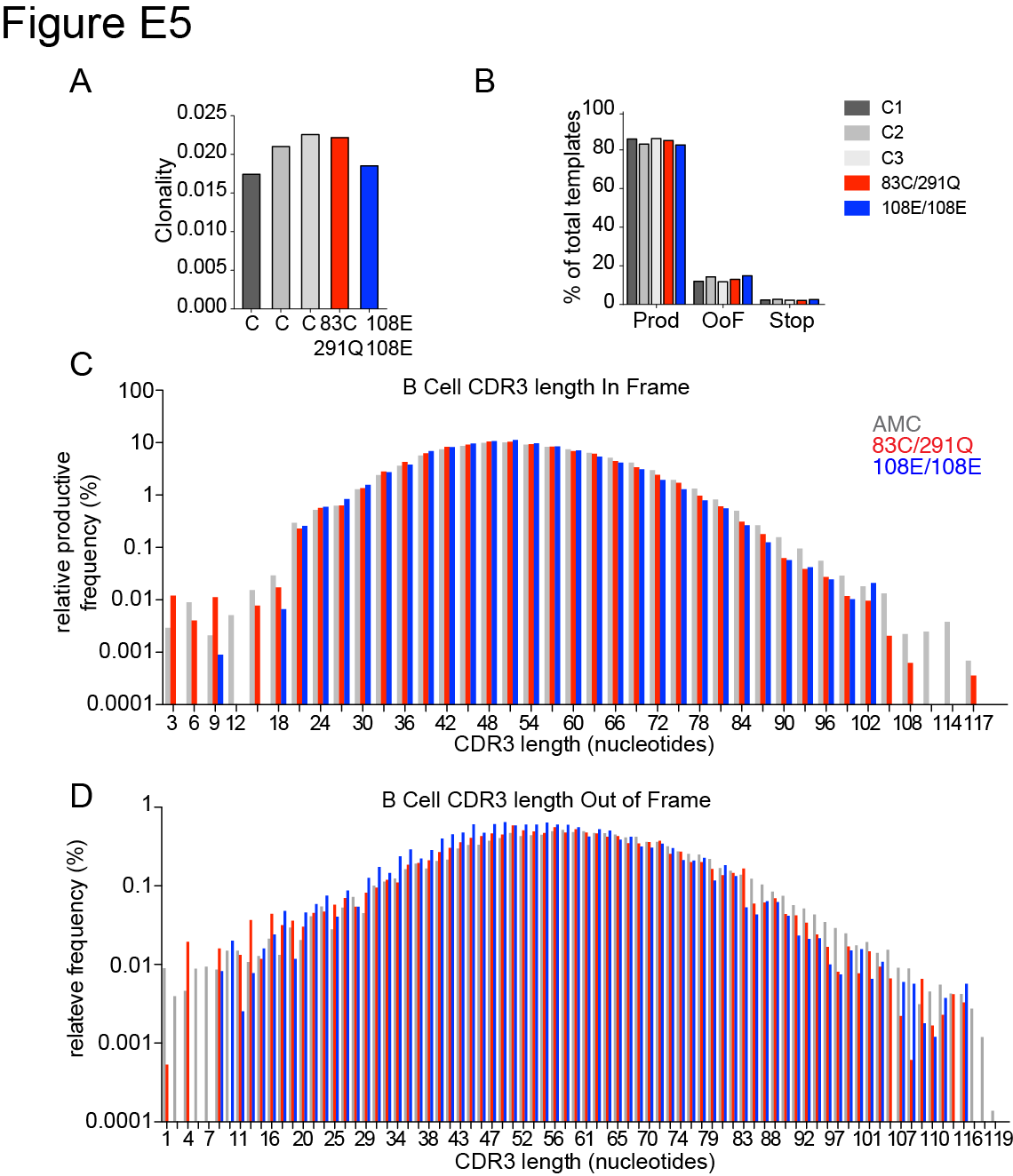
**

### Table E1: Antibodies used for flow cytometry.

Antibody, fluorochrome, clone and manufacturer of antibodies used in flow cytometry

| **Antigen** | **Fluorochrome** | **Clone** | **Manufacturer** |
| --- | --- | --- | --- |
| CCR7 | FITC | G043H7 | Biolegend |
| CD117 | BV605 | 104D2 | BD Biosciences (BD) |
| CD11c | BV421/AF700 | B-ly6 | BD |
| CD123 | PerCP-Cy5.5 | 7G3 | BD |
| CD127 | APC | A019D5 | Biolegend |
| CD14 | BV650 | M5E2 | Biolegend |
| CD141 | APC | AD5-14H12 | Miltenyi |
| CD10 | BV650 | HI10A | BD |
| CD15 | FITC | MMA | BD |
| CD159a | APC | Z199 | BeckmanCoulter |
| CD16 | PE-CF-594/APC-H7//PE-Dazzle-594/PE-Cy7 | 3G8/3G8/3G8/B73.1 | BD/BD/Biolegend/BD |
| CD19 | PE-CF-594/PE/FITC | HIB19/HIB19/4G7 | BD/BD/BD |
| CD1c | PE-Cy7 | L161 | Biolegend |
| CD206 | PE | 19.2 | BD |
| CD21 | APC | B-Ly4 | BD |
| CD24 | FITC | ML5 | BD |
| CD25 | BV650 | BC96 | Biolegend |
| CD27 | BV421/V500 | O323/M-T271 | Biolegend/BD |
| CD3 | FITC/PerCP-Cy5.5/APC-H7/PE | SK7(Leu4)/SK7(Leu4)/SK7/SK7(Leu9) | BD/BD/BD/BD |
| CD34 | BV605/APCCy7 | 581 | Biolegend |
| CD38 | PE-Cy7 | HB7 | BD |
| CD4 | PE/BV420 | SK3 (Leu3a)/RPA-T4 | BD/Biolegend |
| CD45 | AF700/V500 | HI30 | Biolegend/BD |
| CD45RA | BV510 | HI100 | Biolegend |
| CD5 | PE | UCHT2 | Biolegend |
| CD56 | FITC/APC | NCAM16.2 | BD |
| CD57 | eF450 | TB01 | eBioscience |
| CD69 | PE-Cy7 | FN50 | BD |
| CD8 | APC-Cy7 | SK1 | BD |
| CD90 | PerCP-Cy5.5 | 5.00E+10 | Biolegend |
| CXCR3 | PE-Cy7 | 1C6/CXCR3 | BD |
| HLA-DR | V500/AF700 | G46-6 | BD |
| IFNg | APC-Cy7 | 4S.B3 | Biolegend |
| IgD | APC-H7 | IA6-2 | BD |
| IgM | PerCP-Cy5.5 | G20-127 | BD |
| IL-17a | APC | eBio64CAP17 | eBioscience |
| IL-22 | PE | 142928 | R&D Systems |
| IL-4 | PE-Cy7 | 8D4-8 | eBioscience |
| IRF8 | PerCP-eFluor710 | V3GYWCH | eBioscience |
| KIR1, 2, 3 | PE | EB6B/GL183/Z27.3.7 | BeckmanCoulter |
| Lang | PE | DCGM4 | BeckmanCoulter |
| NKG2D | PerCP-Cy5.5 | 1D11 | BD |

### Table E2: Blood profile at age 4yrs

Complete blood count, lymphocyte subsets and routine clinical biochemistry of the subject at age 4yrs. Abbreviations: Hb: hemoglobin; Plt: platelet; MCV: mean cell volume; WBC: white blood count; Neut: neutrophils; Lymph: lyphocytes; Monos: monocytes; Eos: eosinophils; Baso: basophils; Ur: urea; Cr: creatinine; CRP: C-reactive protein; Alb: albumin; Adj: adjusted; Phos: phosphate; bili: bilirubin; ALP: alkaline phosphatase; ALT: alanine transferase; GGT: gamma glutamyl transferase

| **CBC** |  | **Biochemistry** |  |
| --- | --- | --- | --- |
| Hb (115-155) g/L | 112 | Na (133-146) mmol/L | 138 |
| Plt (200-450) 10*9/L | 243 | K (3.5-5) mmol/L | 4 |
| MCV (77-87) fL | 74.8 | Ur (2.5-6.5) mmol/L | 5 |
| WBC (5.5-14.5) 10*6/L | 43.3 | Cr (0-42) mmol/L | 19 |
| Neuts (1.5-8) 10*6/L | 35.9 | CRP (0-5) | 5 |
| Lymphs (1.5-7) 10*6/L | 4.33 | Alb (30-50) g/L | 37 |
| Monos (0.7-1.5) 10*6/L | 0.87 | Adj Ca (2.18-2.63) mol/L | 2.28 |
| Eos (0.3-0.8) 10*6/L | 2.17 | Phos (0.9-1.8) mmol/L | 1.33 |
| Baso (0-0.1) 10*6/L | 0 | Mg (0.7-1) mmol/L | 0.95 |
| **Lymphocytes cells/ml)** |  | Bili (0-21) umol/L | 3 |
| T cells (900-4500) | 2058 | ALP (150-375) U/L | 151 |
| B cells (200-2100) | 1225 | ALT (0-40) U/L | 9 |
| NK Cells (100-1000) | 1458 | GGT (0-70) U/L | 12 |
| CD4+ T cells (500-2400) | 1278 |  |  |
| CD8+ T cells (300-1600) | 740 |  |  |
| CD4:CD8 ratio (0.9-2.9) | 1.73 |  |  |

### Table E3: Serum immunoglobulins and specific antibodies

Serum immunoglobulin isotype concentrations and specific antibodies in the subject at given ages. Abbreviations: Hep, hepatitis; CMV, cytomegalovirus; EBV, Epstein-Barr virus; VCA, viral capsid antigen; HSV, herpes simplex virus; VZV, varicella zoster virus.

| **Age** | **0.5yrs** | **0.75yrs** | **2yrs** | **3yrs** | **3.6yrs** | **Ref range (g/l)** |
| --- | --- | --- | --- | --- | --- | --- |
| IgG | 4.9 | 4 | 10.4 |  | 11.4 | 3.0-9.0 |
| IgA | <0.2 | 0.07 | 0.15 |  | 0.32 | 0.15-0.70 |
| IgM | 0.69 | 0.73 | 1.2 |  | 1.46 | 0.4-1.60 |
| IgE | 4 |  | 23.2 |  |  | 0.5-52 ku/l |
| IgG1 | 4.11 |  |  |  |  | 2.3-5.8 |
| IgG2 | 0.41 |  |  |  |  | 0.3-3.9 |
| IgG3 | 0.44 |  |  |  |  | 0.10-0.80 |
| IgG4 | <0.02 |  |  |  |  | <0.50 |
| Tetanus |  | 0.91 |  | 0.5 |  | adequate >0.15 |
| Hib |  | 3.85 |  | 0.07 |  | adequate >1.0 |
| Pneumococcus |  | 32.6 |  | 12 |  | adequate >30 |
| Hep B IgG |  |  |  |  | Neg |  |
| Hep C IgG |  |  |  |  | Neg |  |
| CMV IgG |  |  |  |  | Neg |  |
| EBV VCA IgG |  |  |  |  | Neg |  |
| Measles IgG |  |  |  |  | Neg |  |
| HSV IgG |  |  |  |  | Neg |  |
| VZV IgG |  |  |  |  | Neg |  |
| Toxoplasma IgG |  |  |  |  | Neg |  |

### Table E4: HuProt™ protein clones

Clones of proteins used in the HuProt™ protein microarray analysis to determine serum auto-reactivity of subject and age-matched controls

| **Name** | **Clone** | **Name** | **Clone** | **Name** | **Clone** | **Name** | **Clone** |
| --- | --- | --- | --- | --- | --- | --- | --- |
| IGHG1 | JHU08300.P087A06 | IGK | JHU05320.P056E07 | KV205 | JHU03021.P032B02 | SIRPB1 | JHU15174.P159B06 |
| IGHG1 | JHU01487.P016B05 | IGKC | JHU15230.P160A07 | KV205 | JHU03021.P186H06 | SNX33 | JHU08338.P087A05 |
| IGHG1 | JHU15996.P168D09 | IGKC | JHU08867.P093C02 | IGKV (frag) | JHU08869.P093A02 | GNB3 | JHU07809.P082C05 |
| IGHG1 | JHU01386.P015B05 | IGKC | JHU15228.P160C12 | IGKV1OR2 | JHU19024.P193G01 | VDAC1 | JHU02868.P030C02 |
| IGHG1 | JHU15225.P160A01 | IGKC | JHU16431.P173A07 | IGL | JHU15249.P160E12 | TMEM116 | JHU01814.P019C02 |
| IGHG1 | JHU15223.P160A12 | IGKC | JHU15137.P159A08 | IGL | JHU08200.P086C07 | KJ900884 (frag) | JHU07737.P081C10 |
| IGHG1 | JHU07812.P082B07 | IGKC | JHU08868.P093C01 | IGL | JHU16434.P173A08 | SCLT1 | JHU15264.P160C07 |
| IGHG1 | JHU02822.P030C03 | IGKC | JHU16293.P171B08 | IGL | JHU08870.P093F02 | SPATC1 | JHU16238.P170A06 |
| IGHG1 | JHU06762.P071F01 | IGKC | JHU16433.P173C08 | IGL | JHU03695.P039F03 | VRK2 | JHU15471.P082B08 |
| IGHG1 | JHU15226.P160C01 | IGKC | JHU16432.P173D07 | IGL | JHU08871.P093E02 | MARCH2 | JHU01787.P019D04 |
| IGHG1 | JHU15993.P168H02 | IGKC | JHU15231.P160C03 | IGL | JHU16435.P173D09 | FN3K | JHU14546.P153F11 |
| IGHG1 | JHU16425.P173C09 | IGKC | JHU09354.P098A07 | IGL | JHU15134.P159C06 | OTUD5 | JHU15158.P159F11 |
| IGHG1 | JHU15224.P160D07 | IGKC | JHU03585.P038A11 | IGL | JHU15135.P159C07 | DNAJC11 | JHU07801.P082A07 |
| IGHG1 | JHU15997.P168F09 | IGKC | JHU15131.P159E07 | IGL | JHU16294.P171A06 | GNA11 | JHU15218.P160A02 |
| IGHG1 | JHU15130.P159H12 | IGKC | JHU08866.P093B02 | IGL | JHU16000.P168G09 | NUDT6 | JHU00927.P010D03 |
| IGHG1 | JHU16423.P173H04 | IGKC | JHU16098.P169H04 | IGL | JHU15328.P161D01 | TSSK1B | JHU00856.P009D08 |
| IGHG1 | JHU15995.P168C09 | IGKC | JHU02036.P189D07 | IGL | JHU15329.P161G04 | GDPD5 | JHU01374.P015H04 |
| IGHG1 | JHU15128.P159G06 | IGKC | JHU06466.P068C09 | IGL | JHU16436.P173E01 | ELK2 (frag) | JHU15432.P162B12 |
| IGHG1 | JHU15994.P180G07 | IGKC | JHU16430.P173G06 | IGL | JHU08871.P093D02 | SESTD1 | JHU07568.P079B10 |
| IGHG1 | JHU15994.P179G07 | IGKC | JHU15229.P160F12 | IGL | JHU15136.P159G07 | IFI35 | JHU00322.P004A07 |
| IGHG1 | JHU08301.P087D10 | IGKC | JHU15227.P160G12 |  |  | SCAMP2 | JHU00364.P004B07 |
| IGHG1 | JHU15327.P161B07 | IGKC | JHU15233.P160H03 |  |  | EIF3D | JHU02720.P029E03 |
| IGHG1 | JHU01874.P189D02 | IGKC | JHU15232.P160D03 |  |  | OCEL1 | JHU08893.P093B01 |
| IGHG1 | JHU06761.P071D01 | IGKC | JHU15132.P159H07 |  |  | APMAP | JHU02415.P026C08 |
| IGHG1 | JHU06646.P070H07 | IGKC | JHU15133.P159F07 |  |  | C17orf62 | JHU02511.P027D11 |
| IGHG1 | JHU15129.P159F10 | IGKC | JHU02823.P030H07 |  |  |  |  |
| IGHG1 | JHU00912.P010G07 | IGKC | JHU16098.P165A04 |  |  |  |  |
| IGHG1 | JHU07813.P082E07 | IGKC | JHU02036.P022B05 |  |  |  |  |
| IGHG1 | JHU01385.P015A12 |  |  |  |  |  |  |
| IGHG1 | JHU16426.P173B01 |  |  |  |  |  |  |
| IGHG3 | JHU08684.P091A07 |  |  |  |  |  |  |
| IGHG3 | JHU15998.P168B09 |  |  |  |  |  |  |
| IGHG4 | JHU03305.P035F12 |  |  |  |  |  |  |
| IGHV5-78 | JHU03793.P040F08 |  |  |  |  |  |  |
| IGHV7-81 | JHU14209.P149E04 |  |  |  |  |  |  |
| IGHD | JHU15222.P160B12 |  |  |  |  |  |  |
| IGHD | JHU00710.P008F07 |  |  |  |  |  |  |
| IGHA1 | JHU16422.P173F01 |  |  |  |  |  |  |
| IGHA1 | JHU15126.P159D07 |  |  |  |  |  |  |
| IGHA1 | JHU16097.P169C08 |  |  |  |  |  |  |
| IGHA1 | JHU05319.P056E09 |  |  |  |  |  |  |
| IGHA1 | JHU04166.P044B02 |  |  |  |  |  |  |
| IGHA1 | JHU09164.P096H06 |  |  |  |  |  |  |
| IGHA2 | JHU15326.P161E03 |  |  |  |  |  |  |
| IGHA2 | JHU16300.P171E11 |  |  |  |  |  |  |

### Table E5: Summary of Whole Exome Sequencing results

Details of the four genes containing bi-allelic mutations to fit an autosomal recessive model of inheritance. Location of the mutations is given, with impact prediction (Sift, Polyphen2, Condel), CADD scores, identification in sequencing databases (dbSNP147, EVS ALL, ExAC) and gene function. Abbreviations: pos: amino acid position; AA: amino acids; del: deleterious; tol: tolerated; Prob D: probably damaging; B: benign; neut: neutral; CADD: Combined Annotation Dependent Depletion; CAF: complete allele frequency; EVS: Exome Variant Server; ExAC: Exome Aggregation Consortium.

| **Symbol** | **Transcript feature** | **Allele** | **Variant** | **Pos** | **AA** | **codons** | **exon** | **Sift** | **Polyphen2** | **Condel** | **CADD Score** |
| --- | --- | --- | --- | --- | --- | --- | --- | --- | --- | --- | --- |
| **IRF8** | ENST00000268638 | A | missense | 291 | R/Q | cGg/cAg | 7/9 | del (0) | Prob D (0.997) | del (0.911) | 36 |
| **IRF8** | ENST00000268638 | T | missense | 83 | R/C | Cgc/Tgc | 3/9 | del (0) | Prob D (0.999) | del (0.935) | 27.8 |
| **TTN** | ENST00000360870 | T | missense | 483 | A/T | Gcc/Acc | 9/46 | - | B (0.181) | - | 14.19 |
| **TTN** | ENST00000360870 | A | missense | 1890 | R/C | Cgc/Tgc | 28/46 | - | Prob D (0.996) | - | 11.01 |
| **CSPG4** | ENST00000308508 | T | missense | 379 | G/S | Ggc/Agc | 3/10 | tol (0.08) | Poss D (0.734) | del (0.569) | 16.24 |
| **CSPG4** | ENST00000308508 | C | missense | 247 | N/S | aAt/aGt | 3/10 | del (0) | Prob D (0.999) | del (0.935) | 15.29 |
| **OR6N1** | ENST00000335094 | T | missense | 7 | S/N | aGc/aAc | 1/1 | tol (0.05) | B (0.035) | neut (0.347) | 14.28 |
| **OR6N1** | ENST00000335094 | T | missense | 219 | V/M | Gtg/Atg | 1/1 | tol (0.11) | B (0.089) | neut (0.278) | 1.09 |

| **Symbol** | **Chromosome position** | **SNP ID** | **dbSNP147_CAF** | **EVS ALL AF** | **ExAC AF NFE** | **Gene and function** |
| --- | --- | --- | --- | --- | --- | --- |
| **IRF8** | 16; 85952293 | - | - | - | - | **IRF8** |
| **IRF8** | 16; 85942668 | - | - | - | - |  |
| **TTN** | 2; 179658220 | rs34337578 | - | - | 6.00078E-05 | **Titin**: a large abundant protein of striated muscle, mutated in familial hypertrophic cardio-myopathy 9 |
| **TTN** | 2; 179640923 | rs146496197 | 0.000599 | 0.00107643 | 0.000179813 |  |
| **CSPG4** | 15; 75982271 | rs141212136 | - | - | 0.00420603 | **Chondroitin sulphate proteoglycan 4:** an integral membrane protein expressed on human malignant melanoma cells |
| **CSPG4** | 15; 75980521 | - | - | - | - |  |
| **OR6N1** | 1; 158736453 | - | - | - | - | **Olfactory Receptor Family 6 Subfamily N Member 1** |
| **OR6N1** | 1; 158735818 | rs368576348 | - | 0.000153775 | 5.99377E-05 |  |

### Table E6: Pathway annotations for differentially expressed genes

Summary of gene ontology (GO) terms identified as significantly enriched (p<0.01) after hyper-geometric testing differentially regulated transcripts as described in Figure S3.

| **CELL TYPE** | **GOBPID** | **P VALUE** | **Hits in DE gene lists** | **Hits in Nano panel** | **TERM** |
| --- | --- | --- | --- | --- | --- |
| **UP in Neutrophils** | GO:0016045 | 0.002222326 | 4 | 9 | detection of bacterium |
|  | GO:0098543 | 0.003511831 | 4 | 10 | detection of other organism |
|  | GO:0051606 | 0.01750708 | 4 | 15 | detection of stimulus |
|  | GO:0002227 | 0.005914357 | 3 | 6 | innate immune response in mucosa |
|  | GO:0002385 | 0.021320939 | 3 | 9 | mucosal immune response |
|  | GO:0050832 | 0.037848665 | 3 | 11 | defense response to fungus |
|  | GO:0032490 | 0.043814273 | 2 | 5 | detection of molecule of bacterial origin |
| **DOWN in Neutrophils** | GO:0019722 | 0.04642981 | 6 | 23 | calcium-mediated signaling |
|  | GO:0031343 | 0.037845887 | 6 | 22 | positive regulation of cell killing |
|  | GO:0001912 | 0.018476463 | 6 | 19 | positive regulation of leukocyte mediated cytotoxicity |
|  | GO:0070228 | 0.018476463 | 6 | 19 | regulation of lymphocyte apoptotic process |
|  | GO:0033628 | 0.00339839 | 5 | 10 | regulation of cell adhesion mediated by integrin |
|  | GO:0050848 | 0.044890105 | 4 | 12 | regulation of calcium-mediated signaling |
|  | GO:0034123 | 0.040849279 | 3 | 7 | positive regulation of toll-like receptor signaling pathway |
|  | GO:0072604 | 0.040849279 | 3 | 7 | interleukin-6 secretion |
|  | GO:0033630 | 0.013957043 | 3 | 5 | positive regulation of cell adhesion mediated by integrin |
|  | GO:0033631 | 0.013957043 | 3 | 5 | cell-cell adhesion mediated by integrin |
|  | GO:0045085 | 0.039491547 | 2 | 3 | negative regulation of interleukin-2 biosynthetic process |
| **UP in B cells** | GO:0002684 | 0.017596268 | 18 | 291 | positive regulation of immune system process |
|  | GO:0009617 | 0.03412948 | 9 | 154 | response to bacterium |
|  | GO:0098542 | 0.005561691 | 9 | 118 | defense response to other organism |
|  | GO:0002683 | 0.004999421 | 8 | 94 | negative regulation of immune system process |
|  | GO:0032496 | 0.049689683 | 7 | 112 | response to lipopolysaccharide |
|  | GO:0050663 | 0.021072203 | 6 | 73 | cytokine secretion |
|  | GO:0001818 | 0.016093398 | 6 | 69 | negative regulation of cytokine production |
|  | GO:0050777 | 0.007877398 | 5 | 42 | negative regulation of immune response |
|  | GO:0002698 | 0.003468525 | 5 | 35 | negative regulation of immune effector process |
|  | GO:1903556 | 0.010132174 | 3 | 15 | negative regulation of tumor necrosis factor superfamily cytokine |
|  | GO:0034121 | 0.008273739 | 3 | 14 | regulation of toll-like receptor signaling pathway |
|  | GO:0043409 | 0.045672724 | 2 | 11 | negative regulation of MAPK cascade |
|  | GO:0050832 | 0.045672724 | 2 | 11 | defense response to fungus |
|  | GO:0019730 | 0.038093783 | 2 | 10 | antimicrobial humoral response |
|  | GO:0032703 | 0.038093783 | 2 | 10 | negative regulation of interleukin-2 production |
|  | GO:0019731 | 0.031067207 | 2 | 9 | antibacterial humoral response |
|  | GO:0032753 | 0.031067207 | 2 | 9 | positive regulation of interleukin-4 production |
|  | GO:0034123 | 0.018835046 | 2 | 7 | positive regulation of toll-like receptor signaling pathway |
|  | GO:0019732 | 0.002907265 | 2 | 3 | antifungal humoral response |
| **DOWN in B cells** | GO:0001819 | 0.031462576 | 11 | 149 | positive regulation of cytokine production |
|  | GO:0000165 | 0.00577869 | 11 | 120 | regulation of MAPK cascade |
|  | GO:0043410 | 0.02274156 | 9 | 105 | positive regulation of MAPK cascade |
|  | GO:0010629 | 0.043425774 | 8 | 98 | negative regulation of gene expression |
|  | GO:0009890 | 0.008159741 | 9 | 90 | negative regulation of biosynthetic process |
|  | GO:0070374 | 0.002877251 | 7 | 49 | positive regulation of ERK1 and ERK2 cascade |
|  | GO:0032733 | 0.043252057 | 3 | 19 | positive regulation of interleukin-10 production |
|  | GO:0032757 | 0.043252057 | 3 | 19 | positive regulation of interleukin-8 production |
|  | GO:0000060 | 0.00926014 | 3 | 11 | protein import into nucleus, translocation |
|  | GO:0042033 | 0.042535558 | 2 | 8 | chemokine biosynthetic process |
|  | GO:0050755 | 0.042535558 | 2 | 8 | chemokine metabolic process |
| **Up in NK cells** | GO:0009620 | 0.029872817 | 4 | 21 | response to fungus |
|  | GO:0032757 | 0.021062229 | 4 | 19 | positive regulation of interleukin-8 production |
|  | GO:0002227 | 0.043980484 | 2 | 6 | innate immune response in mucosa |
|  | GO:0002523 | 0.043980484 | 2 | 6 | leukocyte migration involved in inflammatory response |
|  | GO:0090196 | 0.030441746 | 2 | 5 | regulation of chemokine secretion |
|  | GO:0019732 | 0.009848599 | 2 | 3 | antifungal humoral response |
|  | GO:0060340 | 0.009848599 | 2 | 3 | positive regulation of type I interferon-mediated signaling pathway |
| **DOWN in NK cells** | GO:0006952 | 0.035815526 | 14 | 392 | defense response |
|  | GO:0007166 | 0.038153717 | 13 | 347 | cell surface receptor signaling pathway |
|  | GO:0019222 | 0.034816385 | 13 | 344 | regulation of metabolic process |
|  | GO:0034097 | 0.035519791 | 10 | 226 | response to cytokine |
|  | GO:0031349 | 0.019692748 | 8 | 144 | positive regulation of defense response |
|  | GO:0002757 | 0.026158083 | 7 | 121 | immune response-activating signal transduction |
|  | GO:0009615 | 0.024536277 | 6 | 92 | response to virus |
|  | GO:0034341 | 0.005058186 | 6 | 67 | response to interferon-gamma |
|  | GO:0002819 | 0.014707289 | 5 | 59 | regulation of adaptive immune response |
|  | GO:0002221 | 0.011776317 | 5 | 56 | pattern recognition receptor signaling pathway |
|  | GO:0002224 | 0.007846035 | 5 | 51 | toll-like receptor signaling pathway |
|  | GO:0001959 | 0.020746086 | 4 | 42 | regulation of cytokine-mediated signaling pathway |
|  | GO:0034138 | 0.048907012 | 3 | 32 | toll-like receptor 3 signaling pathway |
|  | GO:0032663 | 0.041374314 | 3 | 30 | regulation of interleukin-2 production |
|  | GO:0034340 | 0.041374314 | 3 | 30 | response to type I interferon |
|  | GO:0034134 | 0.034503322 | 3 | 28 | toll-like receptor 2 signaling pathway |
|  | GO:0034146 | 0.015719153 | 3 | 21 | toll-like receptor 5 signaling pathway |
|  | GO:0033673 | 0.010132174 | 3 | 18 | negative regulation of kinase activity |
|  | GO:0045620 | 0.044509817 | 2 | 13 | negative regulation of lymphocyte differentiation |
|  | GO:0060330 | 0.026912182 | 2 | 10 | regulation of response to interferon-gamma |
|  | GO:0060334 | 0.026912182 | 2 | 10 | regulation of interferon-gamma-mediated signaling pathway |
|  | GO:1904029 | 0.021869613 | 2 | 9 | regulation of cyclin-dependent protein kinase activity |
| **UP in T cells** | GO:0019725 | 0.035980664 | 10 | 75 | cellular homeostasis |
|  | GO:0050832 | 0.005737437 | 4 | 11 | defense response to fungus |
|  | GO:0034123 | 0.010551681 | 3 | 7 | positive regulation of toll-like receptor signaling pathway |
|  | GO:0034145 | 0.015142508 | 2 | 3 | positive regulation of toll-like receptor 4 signaling pathway |
|  | GO:0002430 | 0.005295584 | 2 | 2 | complement receptor mediated signaling pathway |
| **DOWN in T cells** | GO:0010629 | 0.000984353 | 11 | 98 | negative regulation of gene expression |
|  | GO:0002221 | 0.025335714 | 6 | 56 | pattern recognition receptor signaling pathway |
|  | GO:0002224 | 0.016293373 | 6 | 51 | toll-like receptor signaling pathway |
|  | GO:0031098 | 0.027738514 | 5 | 42 | stress-activated protein kinase signaling cascade |
|  | GO:0032640 | 0.022768595 | 5 | 40 | tumor necrosis factor production |
|  | GO:0034142 | 0.022768595 | 5 | 40 | toll-like receptor 4 signaling pathway |
|  | GO:0034138 | 0.008831489 | 5 | 32 | toll-like receptor 3 signaling pathway |
|  | GO:0031663 | 0.004850777 | 5 | 28 | lipopolysaccharide-mediated signaling pathway |
|  | GO:0035666 | 0.027070881 | 4 | 28 | TRIF-dependent toll-like receptor signaling pathway |
|  | GO:0042770 | 0.01153942 | 4 | 22 | signal transduction in response to DNA damage |
|  | GO:0034146 | 0.009723364 | 4 | 21 | toll-like receptor 5 signaling pathway |
|  | GO:0034166 | 0.04945423 | 3 | 20 | toll-like receptor 10 signaling pathway |
|  | GO:0072678 | 0.032136155 | 3 | 17 | T cell migration |
|  | GO:1902806 | 0.032136155 | 3 | 17 | regulation of cell cycle G1/S phase transition |
|  | GO:0032720 | 0.0151558 | 3 | 13 | negative regulation of tumor necrosis factor production |
|  | GO:0042771 | 0.00693088 | 3 | 10 | intrinsic apoptotic signaling pathway in response to DNA damage |
|  | GO:0042090 | 0.02402828 | 2 | 6 | interleukin-12 biosynthetic process |
|  | GO:0045075 | 0.02402828 | 2 | 6 | regulation of interleukin-12 biosynthetic process |
|  | GO:0031032 | 0.01645086 | 2 | 5 | actomyosin structure organization |
|  | GO:0032692 | 0.01645086 | 2 | 5 | negative regulation of interleukin-1 production |
|  | GO:0045084 | 0.01645086 | 2 | 5 | positive regulation of interleukin-12 biosynthetic process |
| **Up in NK cells** | GO:0009620 | 0.029872817 | 4 | 21 | response to fungus |
|  | GO:0032757 | 0.021062229 | 4 | 19 | positive regulation of interleukin-8 production |
|  | GO:0002227 | 0.043980484 | 2 | 6 | innate immune response in mucosa |
|  | GO:0002523 | 0.043980484 | 2 | 6 | leukocyte migration involved in inflammatory response |
|  | GO:0090196 | 0.030441746 | 2 | 5 | regulation of chemokine secretion |
|  | GO:0019732 | 0.009848599 | 2 | 3 | antifungal humoral response |
|  | GO:0060340 | 0.009848599 | 2 | 3 | positive regulation of type I interferon-mediated signaling pathway |
| **DOWN in NK cells** | GO:0006952 | 0.035815526 | 14 | 392 | defense response |
|  | GO:0007166 | 0.038153717 | 13 | 347 | cell surface receptor signaling pathway |
|  | GO:0019222 | 0.034816385 | 13 | 344 | regulation of metabolic process |
|  | GO:0034097 | 0.035519791 | 10 | 226 | response to cytokine |
|  | GO:0031349 | 0.019692748 | 8 | 144 | positive regulation of defense response |
|  | GO:0002757 | 0.026158083 | 7 | 121 | immune response-activating signal transduction |
|  | GO:0009615 | 0.024536277 | 6 | 92 | response to virus |
|  | GO:0034341 | 0.005058186 | 6 | 67 | response to interferon-gamma |
|  | GO:0002819 | 0.014707289 | 5 | 59 | regulation of adaptive immune response |
|  | GO:0002221 | 0.011776317 | 5 | 56 | pattern recognition receptor signaling pathway |
|  | GO:0002224 | 0.007846035 | 5 | 51 | toll-like receptor signaling pathway |
|  | GO:0001959 | 0.020746086 | 4 | 42 | regulation of cytokine-mediated signaling pathway |
|  | GO:0034138 | 0.048907012 | 3 | 32 | toll-like receptor 3 signaling pathway |
|  | GO:0032663 | 0.041374314 | 3 | 30 | regulation of interleukin-2 production |
|  | GO:0034340 | 0.041374314 | 3 | 30 | response to type I interferon |
|  | GO:0034134 | 0.034503322 | 3 | 28 | toll-like receptor 2 signaling pathway |
|  | GO:0034146 | 0.015719153 | 3 | 21 | toll-like receptor 5 signaling pathway |
|  | GO:0033673 | 0.010132174 | 3 | 18 | negative regulation of kinase activity |
|  | GO:0045620 | 0.044509817 | 2 | 13 | negative regulation of lymphocyte differentiation |
|  | GO:0060330 | 0.026912182 | 2 | 10 | regulation of response to interferon-gamma |
|  | GO:0060334 | 0.026912182 | 2 | 10 | regulation of interferon-gamma-mediated signaling pathway |
|  | GO:1904029 | 0.021869613 | 2 | 9 | regulation of cyclin-dependent protein kinase activity |
| **UP in T cells** | GO:0019725 | 0.035980664 | 10 | 75 | cellular homeostasis |
|  | GO:0050832 | 0.005737437 | 4 | 11 | defense response to fungus |
|  | GO:0034123 | 0.010551681 | 3 | 7 | positive regulation of toll-like receptor signaling pathway |
|  | GO:0034145 | 0.015142508 | 2 | 3 | positive regulation of toll-like receptor 4 signaling pathway |
|  | GO:0002430 | 0.005295584 | 2 | 2 | complement receptor mediated signaling pathway |
| **DOWN in T cells** | GO:0010629 | 0.000984353 | 11 | 98 | negative regulation of gene expression |
|  | GO:0002221 | 0.025335714 | 6 | 56 | pattern recognition receptor signaling pathway |
|  | GO:0002224 | 0.016293373 | 6 | 51 | toll-like receptor signaling pathway |
|  | GO:0031098 | 0.027738514 | 5 | 42 | stress-activated protein kinase signaling cascade |
|  | GO:0032640 | 0.022768595 | 5 | 40 | tumor necrosis factor production |
|  | GO:0034142 | 0.022768595 | 5 | 40 | toll-like receptor 4 signaling pathway |
|  | GO:0034138 | 0.008831489 | 5 | 32 | toll-like receptor 3 signaling pathway |
|  | GO:0031663 | 0.004850777 | 5 | 28 | lipopolysaccharide-mediated signaling pathway |
|  | GO:0035666 | 0.027070881 | 4 | 28 | TRIF-dependent toll-like receptor signaling pathway |
|  | GO:0042770 | 0.01153942 | 4 | 22 | signal transduction in response to DNA damage |
|  | GO:0034146 | 0.009723364 | 4 | 21 | toll-like receptor 5 signaling pathway |
|  | GO:0034166 | 0.04945423 | 3 | 20 | toll-like receptor 10 signaling pathway |
|  | GO:0072678 | 0.032136155 | 3 | 17 | T cell migration |
|  | GO:1902806 | 0.032136155 | 3 | 17 | regulation of cell cycle G1/S phase transition |
|  | GO:0032720 | 0.0151558 | 3 | 13 | negative regulation of tumor necrosis factor production |
|  | GO:0042771 | 0.00693088 | 3 | 10 | intrinsic apoptotic signaling pathway in response to DNA damage by p53 |
|  | GO:0042090 | 0.02402828 | 2 | 6 | interleukin-12 biosynthetic process |
|  | GO:0045075 | 0.02402828 | 2 | 6 | regulation of interleukin-12 biosynthetic process |
|  | GO:0031032 | 0.01645086 | 2 | 5 | actomyosin structure organization |
|  | GO:0032692 | 0.01645086 | 2 | 5 | negative regulation of interleukin-1 production |
|  | GO:0045084 | 0.01645086 | 2 | 5 | positive regulation of interleukin-12 biosynthetic process |

### Table E7: Differentially expressed genes shared in K108E and 83C/291Q

Genes identified as dysregulated (and IRF8-bound in the second column) both in the patient bearing *IRF8^K108E/K108E^* by RNA-Seq of PBMC^14^ and the patient with *IRF8^R83C/R291Q^* by Nanostring nCounter analysis of FACs-purified lymphoid subsets or density centrifugation purification of neutrophils.

| Shared | Shared IRF8 bound |
| --- | --- |
| CAMP | CCL4 |
| CCL3 | CCR5 |
| CCL4 | CD1D |
| CCR5 | CD244 |
| CD19 | CXCR6 |
| CD1D | GPR183 |
| CD24 | LTF |
| CD244 | MALT1 |
| CD28 | MS4A1 |
| CD5 | NFATC2 |
| CD8A | S100A8 |
| CEACAM8 | S1PR1 |
| CXCR6 | SLC2A1 |
| FADD | TAGAP |
| FKBP5 | TGFBI |
| FYN |  |
| GPR183 |  |
| GZMB |  |
| KIR3DL2 |  |
| LILRB4 |  |
| LTF |  |
| MALT1 |  |
| MAPK11 |  |
| MS4A1 |  |
| MUC1 |  |
| NFATC2 |  |
| S100A8 |  |
| S100A9 |  |
| S1PR1 |  |
| SLAMF1 |  |
| SLC2A1 |  |
| TAGAP |  |
| TGFBI |  |
| TIGIT |  |
| TLR3 |  |
| TNFRSF10C |  |

## Supplementary References

1. Li H, Handsaker B, Wysoker A, Fennell T, Ruan J, Homer N, Marth G, Abecasis G, Durbin R, 1000 GPDPS. The Sequence Alignment/Map format and SAMtools. Bioinformatics 2009;25:2078-2079.

2. DePristo MA, Banks E, Poplin R, Garimella KV, Maguire JR, Hartl C, Philippakis AA, del Angel G, Rivas MA, Hanna M, McKenna A, Fennell TJ, Kernytsky AM, Sivachenko AY, Cibulskis K, Gabriel SB, Altshuler D, Daly MJ. A framework for variation discovery and genotyping using next-generation DNA sequencing data. Nat Genet 2011;43:491-498.

3. McKenna A, Hanna M, Banks E, Sivachenko A, Cibulskis K, Kernytsky A, Garimella K, Altshuler D, Gabriel S, Daly M, DePristo MA. The Genome Analysis Toolkit: a MapReduce framework for analyzing next-generation DNA sequencing data. Genome Res 2010;20:1297-1303.

4. McLaren W, Pritchard B, Rios D, Chen Y, Flicek P, Cunningham F. Deriving the consequences of genomic variants with the Ensembl API and SNP Effect Predictor. Bioinformatics 2010;26:2069-2070.

5. Kircher M, Witten DM, Jain P, O’Roak BJ, Cooper GM, Shendure J. A general framework for estimating the relative pathogenicity of human genetic variants. Nat Genet 2014;46:310-315.

6. Malnic B, Godfrey PA, Buck LB. The human olfactory receptor gene family. Proc Natl Acad Sci U S A 2004;101:2584-2589.

7. Foley J, Witte D, Chiu FC, Parysek LM. Expression of the neural intermediate filament proteins peripherin and neurofilament-66/alpha-internexin in neuroblastoma. Lab Invest 1994;71:193-199.

8. Hambleton S, Salem S, Bustamante J, Bigley V, Boisson-Dupuis S, Azevedo J, Fortin A, Haniffa M, Ceron-Gutierrez L, Bacon CM, Menon G, Trouillet C, McDonald D, Carey P, Ginhoux F, Alsina L, Zumwalt TJ, Kong XF, Kumararatne D, Butler K, Hubeau M, Feinberg J, Al-Muhsen S, Cant A, Abel L, Chaussabel D, Doffinger R, Talesnik E, Grumach A, Duarte A, Abarca K, Moraes-Vasconcelos D, Burk D, Berghuis A, Geissmann F, Collin M, Casanova JL, Gros P. IRF8 mutations and human dendritic-cell immunodeficiency. N Engl J Med 2011;365:127-138.

9. Doody GM, Stephenson S, McManamy C, Tooze RM. PRDM1/BLIMP-1 modulates IFN-gamma-dependent control of the MHC class I antigen-processing and peptide-loading pathway. J Immunol 2007;179:7614-7623.

10. Rusinova I, Forster S, Yu S, Kannan A, Masse M, Cumming H, Chapman R, Hertzog PJ. Interferome v2.0: an updated database of annotated interferon-regulated genes. Nucleic Acids Res 2013;41:D1040-6.

11. Langlais D, Barreiro LB, Gros P. The macrophage IRF8/IRF1 regulome is required for protection against infections and is associated with chronic inflammation. J Exp Med 2016;213:585-603.

12. Carlson CS, Emerson RO, Sherwood AM, Desmarais C, Chung MW, Parsons JM, Steen MS, LaMadrid-Herrmannsfeldt MA, Williamson DW, Livingston RJ, Wu D, Wood BL, Rieder MJ, Robins H. Using synthetic templates to design an unbiased multiplex PCR assay. Nat Commun 2013;4:2680.

13. Schatorje EJ, Gemen EF, Driessen GJ, Leuvenink J, van Hout RW, de Vries E. Paediatric reference values for the peripheral T cell compartment. Scand J Immunol 2012;75:436-444.

14. Salem S, Langlais D, Lefebvre F, Bourque G, Bigley V, Haniffa M, Casanova JL, Burk D, Berghuis A, Butler KM, Leahy TR, Hambleton S, Gros P. Functional characterization of the human dendritic cell immunodeficiency associated with the IRF8(K108E) mutation. Blood 2014;124:1894-1904.
